# RING finger 138 deregulation distorts NF-кB signaling and facilities colitis switch to aggressive malignancy

**DOI:** 10.1038/s41392-022-00985-1

**Published:** 2022-06-13

**Authors:** Yalan Lu, Rong Huang, Jianming Ying, Xingchen Li, Tao Jiao, Lei Guo, Haitao Zhou, Han Wang, Amannisa Tuersuntuoheti, Jianmei Liu, Qichen Chen, Yanhong Wang, Luying Su, Changyuan Guo, Fu Xu, Ziyi Wang, Yan Lu, Kai Li, Junbo Liang, Zhen Huang, Xiao Chen, Jinjie Yao, Hanjie Hu, Xiaowen Cheng, Yufeng Wan, Xinyan Chen, Ning Zhang, Shiying Miao, Jianqiang Cai, Linfang Wang, Changzheng Liu, Wei Song, Hong Zhao

**Affiliations:** 1 Department of Biochemistry and Molecular Biology, State Key Laboratory of Medical Molecular Biology, Institute of Basic Medical Sciences Chinese Academy of Medical Sciences, School of Basic Medicine Peking Union Medical College, Beijing, 100005 China; 2 Department of Hepatobiliary Surgery, State Key Laboratory of Molecular Oncology, National Cancer Center/National Clinical Research Center for Cancer/Cancer Hospital, Chinese Academy of Medical Sciences and Peking Union Medical College, Beijing, 100021 China; 3 Key Laboratory of Gene Editing Screening and R&D of Digestive System Tumor Drugs, Chinese Academy of Medical Sciences and Peking Union Medical College, Beijing, 100021 China; 4 Key Laboratory of Human Disease Comparative Medicine, Chinese Ministry of Health, Beijing Key Laboratory for Animal Models of Emerging and Remerging Infectious Diseases, Institute of Laboratory Animal Science, Chinese Academy of Medical Sciences and Comparative Medicine Center, Peking Union Medical College, Beijing, 100021 China; 5 National Cancer Center/National Clinical Research Center for Cancer/ Cancer Hospital & Shenzhen Hospital, Chinese Academy of Medical Sciences and Peking Union Medical College, Shenzhen, 518116 China; 6 Department of Pathology, State Key Laboratory of Molecular Oncology, National Cancer Center/National Clinical Research Center for Cancer/Cancer Hospital, Chinese Academy of Medical Sciences and Peking Union Medical College, Beijing, 100021 China; 7 Department of Clinical Laboratory, the First Affiliated Hospital, Anhui Medical University, Hefei, 230022 China; 8 Wellcome Centre for Anti-Infectives Research (WCAIR), Division of Biological Chemistry and Drug Discovery, School of Life Sciences, University of Dundee, Dundee, DD1 5EH UK

**Keywords:** Gastrointestinal cancer, Molecular biology, Target identification

## Abstract

Prolonged activation of nuclear factor (NF)-кB signaling significantly contributes to the development of colorectal cancer (CRC). New therapeutic opportunities are emerging from targeting this distorted cell signaling transduction. Here, we discovered the critical role of RING finger 138 (RNF138) in CRC tumorigenesis through regulating the NF-кB signaling, which is independent of its Ubiquitin-E3 ligase activity involved in DNA damage response. RNF138^−/−^ mice were hyper-susceptible to the switch from colitis to aggressive malignancy, which coincided with sustained aberrant NF-кB signaling in the colonic cells. Furthermore, RNF138 suppresses the activation of NF-кB signaling pathway through preventing the translocation of NIK and IKK-Beta Binding Protein (NIBP) to the cytoplasm, which requires the ubiquitin interaction motif (UIM) domain. More importantly, we uncovered a significant correlation between poor prognosis and the downregulation of RNF138 associated with reinforced NF-кB signaling in clinical settings, raising the possibility of RNF138 dysregulation as an indicator for the therapeutic intervention targeting NF-кB signaling. Using the xenograft models built upon either RNF138-dificient CRC cells or the cells derived from the RNF138-dysregulated CRC patients, we demonstrated that the inhibition of NF-кB signaling effectively hampered tumor growth. Overall, our work defined the pathogenic role of aberrant NF-кB signaling due to RNF138 downregulation in the cascade events from the colitis switch to colonic neoplastic transformation and progression, and also highlights the possibility of targeting the NF-кB signaling in treating specific subtypes of CRC indicated by RNF138-ablation.

## Introduction

Colorectal carcinoma (CRC), accounting for the third leading cause of cancer-related death worldwide,^1,2^ often arises from sustained inflammation and presents chronic inflammation throughout their progression. Being etiologically associated with CRC progression, chronic inflammation affects all stages of CRC pathogenesis, from tumor initiation, progression, to metastasis.^3–6^ Recent advance in understanding the underlying mechanisms began to enable the therapeutic interventions through targeting the CRC-associated chronic inflammation.^7,8^

The nuclear factor-kappa B (NF-кB) signaling is central to the response to environmental stress, genotoxicity, or infections.^9–11^ NF-кB signaling is activated *via* two different routes, i.e., the canonical pathway (CP) and the alternative pathway.^12^ The latter operates predominately in the stimulated B cells as opposed to the former general activation mechanism.^13^ The IκB kinase (IKK) complex, constructed by three subunits, IKKα, IKKβ, and IKKγ, is key component of the CP.^14^ Upon the signal activation, IкB proteins become destabilized after a sequential modification process, first being phosphorylated by IKKβ and then ubiquitinated through the designated Ub E2/E3 enzymes, which ultimately releases NF-кB as its binding partner into the nucleus to switch on the transcription of targeted genes.^14,15^ Ubiquitination also plays important roles in the processing of NF-кB precursors as well as the assembly and regulation of the IKK complex.^16–18^ Importantly, growing evidence has linked the dysregulation of NF-кB signaling to various critical medical conditions. In CRC particularly, the aberrant signaling appears to promote the progression of a cascade events from colitis switch to colonic neoplastic transformation.^12,19,20^

Protein ubiquitination serves critical regulatory roles in the NF-κB signaling transduction.^14,21^ RING finger protein 138 (RNF138), as a Ubiquitin-E3 (Ub-E3) ligase, promotes cell survival *via* counteracting the apoptotic signaling as well as directly engaging in maintaining genome stability.^22–24^ With emerging evidence, RNF138 has been associated with tumorigenesis, neurotic degenerative disorders, and chronic inflammatory conditions.^25–28^ In particular, diverse types of cancer display differential expressions of RNF138 as documented in multiple transcriptomic and proteomic studies, although the relevant functional consequences remain largely elusive. Here, we established a close correlation between RNF138 expression and prognosis of CRC patients. Further investigation revealed that deficient RNF138 led to the dysregulation of NF-κB signaling that significantly promotes the colitis switch to malignancy, which can be effectively suppressed with specific inhibitors against individual components of the pathway.

## Results

### Downregulation of RNF138 in CRC tumors correlates with poor prognosis

**Fig. 1:**
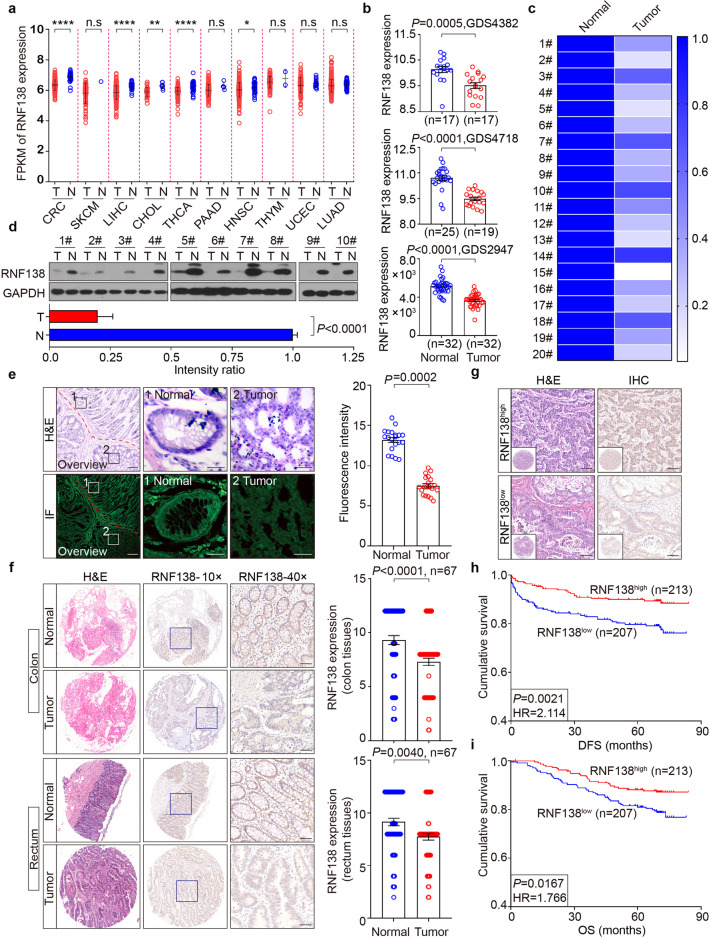
Downregulation of RNF138 in CRC tumors correlates with poor prognosis. *RNF138* mRNA levels in 10 cancer types in the TCGA database (**a**) and three independent GEO datasets (**b**). T tumor tissues, N normal tissues. n.s. not significant; *P < 0.05; **P < 0.01; ****P < 0.0001. **c** Heatmap depiction of *RNF138* mRNA levels assessed by qPCR in 20 CRC samples. **d** Immunoblots and quantification of RNF138 in 10 CRC samples. **e** H&E and IF staining for RNF138 and DAPI in a representative CRC sample. Dashed white line represents the boundary between normal and neoplastic tissues. Histogram (right) shows the quantification of RNF138-staining in CRC or adjacent normal tissues (*n* = 20). Scale bar, 20 μm. **f** H&E and RNF138-staining in 134 CRC tissues (top, colon tissues, *n* = 67; bottom, rectum tissues, *n* = 67). Histograms show quantification of RNF138-staining in CRC and adjacent normal tissues (right). Scale bar, 100 μm. **g** Representative H&E and RNF138-staining images in high and low RNF138 expression in 420 CRC TMAs. The inset box shows an overview of the specimen staining. Scale bar, 25 μm. Kaplan–Meier survival curves showing the correlation between RNF138 expression and DFS (**h**) or OS (**i**) in 420 CRC samples

In analyzing the data collected from The Cancer Genome Atlas (TCGA) database, we noticed that *RNF138* is downregulated in different subsets of cancers, particularly in CRC, liver hepatocellular carcinoma, and thyroid carcinoma, to a less extent in cholangiocarcinoma and head and neck squamous cell carcinoma (Fig. 1a; Supplementary Table 1). This unique expression pattern was also found when using the data derived from additional independent GEO datasets (GDS4382, GDS4718, and GDS2947) (Fig. 1b). In addition, *RNF138* was confirmed to be downregulated in 20 randomized CRC samples compared with the matched adjacent normal tissues at transcriptional level (Fig. 1c; Supplementary Table 2) as well as at translational level (Fig. 1d) (Supplementary Fig. S1a–c). Similar results were obtained when analyzing additional 5 CRC samples in pairs by immunofluorescence (IF) (Fig. 1e; Supplementary Fig. S1d), consistent with the results from further examining 134 pairs of CRC tissue specimens by immunohistochemistry (IHC) (Fig. 1f; Supplementary Table 3). Therefore, RNF138 downregulation is closely associated with CRC tumorigenesis.

We probed the expression of *RNF138* in tissue microarrays (TMAs) of 420 CRC samples (Supplementary Table 4) with the clinicopathological parameter setup established (Supplementary Fig. S2a). The reduction in *RNF138* expression was found specifically associated with more aggressive tumor phenotypes (*P* = 0.027), reflected by both the increase in the size and extent of primary tumors (*P* = 0.003) and lymph node invasion (*P* = 0.003), while no significant correlation was observed with CRC tumor metastasis (*P* = 0.246) (Supplementary Fig. S2b–d; Supplementary Table 4). Importantly, the Kaplan–Meier survival analysis revealed that the increase in *RNF138* expression closely correlates with enhanced disease-free survival (DFS) and improved overall survival (OS) prognoses of patients (Fig. 1g–i), suggesting RNF138 expression level as a prognosis indicator of CRC.

### Ablation of *RNF138* expression exacerbates the progression from colitis to aggressive malignancy

**Fig. 2:**
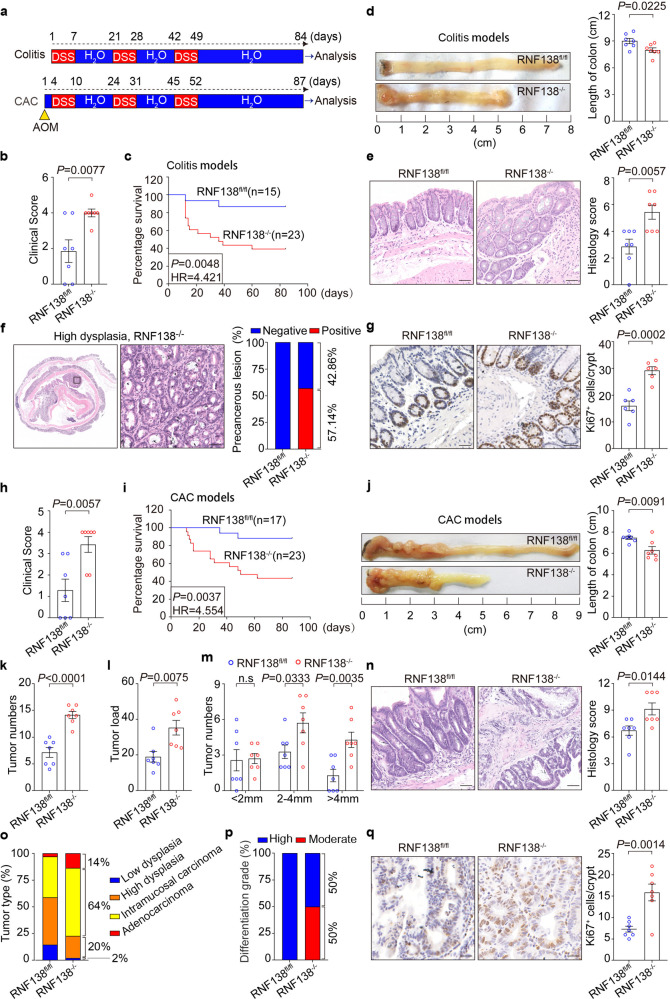
Ablation of *RNF138* expression exacerbates the progression from colitis to aggressive malignancy. **a** Schematic overview of chronic colitis and CAC models. **b** Clinical scores of RNF138^fl/fl^ and RNF138^−/−^ mice (*n* = 7) on the 9th day after DSS administration. **c** Kaplan–Meier survival analysis of RNF138^fl/fl^ (*n* = 15) and RNF138^−/−^ (*n* = 23) mice during DSS treatment. **d** Macroscopic appearance of colons of RNF138^fl/fl^ and RNF138^−/−^ mice (*n* = 7) cohorts at the end of DSS treatment. **e** Representative H&E images (left) with histogram of histology scores (right) of colon tissues for chronic colitis model. Data are mean ± SEM (*n* = 7). Scale bar, 100 μm. **f** Representative H&E staining of precancerous lesions in colitis models. Histogram of precancerous percentages from RNF138^fl/fl^ and RNF138^−/−^ mice after DSS administration (*n* = 7). Scale bar, 100 μm. **g** Representative Ki67-staining (left) and quantification positive cells per crypt (right) in RNF138^fl/fl^ and RNF138^−/−^ colitis models (*n* = 7). Scale bar, 25 μm. **h** Clinical scores of RNF138^fl/fl^ and RNF138^−/−^ mice (*n* = 7) on the 12th day after AOM injection. **i** Kaplan–Meier survival analysis of RNF138^fl/fl^ (*n* = 17) and RNF138^−/−^ (*n* = 23) mice during the course of AOM/DSS treatment. (**j**) Representative longitudinal luminal views of colons from RNF138^fl/fl^ and RNF138^−/−^ mice (*n* = 7) at the end of the AOM/DSS treatment. Tumor numbers (**k**), tumor load (**l**), and tumor size distribution (**m**) of RNF138^fl/fl^ and RNF138^−/−^ mice (*n* = 7) after AOM/DSS treatment. **n** Representative H&E images (left) and histological analysis (right) of colon tissues from RNF138^fl/fl^ and RNF138^−/−^ mice (*n* = 7) after AOM/DSS administration. Scale bar, 100 μm. Percentages of tumor types (**o**) and differentiation grade (**p**) in RNF138^fl/fl^ vs. RNF138^−/−^ mice. **q** Representative Ki67-staining (left) and quantification of positive cells per crypt (right) in RNF138^fl/fl^ and RNF138^−/−^ CAC models (*n* = 7). Scale bar, 25 μm

Chronic inflammation, often in the form of colitis in colorectal tissue, plays important roles in the colonic neoplastic transformation and progression.^6,29^ We next interrogated the possible role of RNF138 in the colitis-associated colorectal tumorigenesis. The chronic colitis and the colitis-associated colorectal cancer (CAC) animal models were developed as illustrated in Fig. 2a. Upon the colitis occurrence, *RNF138*^−/−^ mice deteriorated more rapidly than *RNF138*^fl/fl^ mice under co-housing condition, which was reflected in both the change in clinical scores and weight loss (Fig. 2b; Supplementary Fig. S3a), and ultimately suffered a higher mortality rate (Fig. 2c). The colorectum in *RNF138*^−/−^ mice were significantly reduced in length and exhibited splenomegaly as opposed to the control (Fig. 2d; Supplementary Fig. S3b–d). In *RNF138*^−/−^ mice, extensive inflammation was manifested throughout the mucosa along with reinforced lymphocytic infiltration as revealed by IHC, which coincided with altered epithelial structures and loss of crypts (Fig. 2e). In contrast, only inflammatory lesions were found in the control mice. Furthermore, precancerous lesions in the colorectal tissue were more evident in the *RNF138*^−/−^ (57.14%) (Fig. 2f). Two out of seven *RNF138*^−/−^ mice developed substantial CRC tumors with high-grade dysplasia (Supplementary Fig. S3e). Meanwhile, excessive cell proliferation indicated by Ki67 occurred in the colonic tissues of *RNF138*^−/−^ mice (Fig. 2g).

Similarly, *RNF138*^−/−^ mice exhibited higher clinical scores and suffered more significant weight loss and higher mortality rate during the induction of CAC with azoxymethane (AOM) and dextran sodium sulfate (DSS) (Fig. 2h, i; Supplementary Fig. S3f). In these mice, both colon and spleen tissues were also significantly shortened in length and displayed more severe damage (Fig. 2j; Supplementary Fig. S3g, h). Particularly, all *RNF138*^−/−^ mice (23/23) developed CRC tumors as opposed to 86.7% in the control mice (15/17) (Supplementary Fig. S3i). Furthermore, we found that both the tumor number and tumor load were nearly doubled in the *RNF138*^−/−^ mice (Fig. 2k–l) along with significant increase in size (Fig. 2m). The tumors in these mice appeared to be more advanced (Fig. 2n), developing higher levels of intramucosal carcinoma and adenocarcinoma that displayed lower degree of differentiation (Fig. 2o, p; Supplementary Fig. S3j). Meanwhile, augmented cell proliferation, highlighted by the Ki67-staining, was also identified in the *RNF138*^−/−^ mice tissues (Fig. 2q). Thus, we propose that RNF138 suppresses the chronic colitis switch to colonic neoplastic transformation.

### *RNF138* deletion reinforces tumorigenesis

**Fig. 3:**
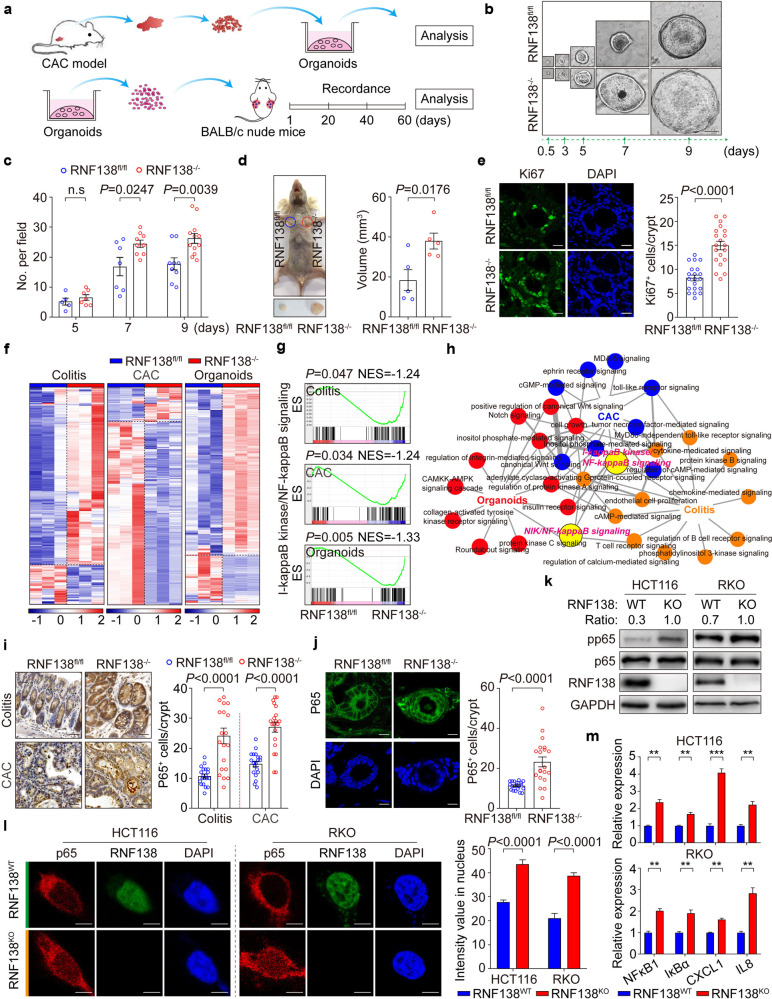
RNF138 inhibits chronic colitis and CRC tumorigenesis *via* NF-кB signaling. **a** Schematic overview of in vitro organoid culture of primary mouse colorectal tumors (top) and xenografts (bottom). **b** Representative images of mouse tumor organoids at different time-points. Scale bar, 200 μm. **c** Quantification of mouse intestinal tumor organoid numbers per field from the 5th to 9th day. **d** Representative images of xenograft tumor organoids in RNF138^fl/fl^ and RNF138^−/−^ mice (*n* = 5) on the 60th day after implantation (left). Quantification of the tumor volume at endpoint (right). **e** Representative images for Ki67-staining and DAPI of xenograft tumor organoids (left). Quantification of number of Ki67-positive cells per crypt (*n* = 20, right). Scale bar, 20 μm. **f** Heatmap depiction of differentially expressed genes in RN138^fl/fl^ and RNF138^−/−^ colorectal cells. **g** GSEA of NF-kappa B signaling relative to RNF138^−/−^ against RNF138^fl/fl^ mice in colitis, CAC, and organoid models. Enrichment plots are shown along with the normalized enrichment score and *P* value. **h** Protein-protein interaction analysis of significant GO terms in colitis, CAC, and organoid models. Circles at nodes represent GO terms in one model (orange, colitis; blue, CAC; red, organoid model; yellow, I-κB kinase/NF-κB, and NIK/NF-κB signaling). **i** Representative p65-staining of colorectal sections from RNF138^fl/fl^ and RNF138^−/−^ mouse colitis (top) and CAC (bottom) models. Quantification of nuclear p65-positive cells per crypt (*n* = 20) (right). Scale bar, 25 μm. **j** Representative images of p65-immunostaining and DAPI from xenograft tumor organoids (left). Quantification of nuclear p65-positive cells per crypt (*n* = 20) (right). Scale bar, 20 μm. **k** Immunoblots of phosphorylation and total levels of p65 in RNF138^WT^ and RNF138^KO^ CRC cells. **l** Representative images of p65-immunostaining in RNF138^WT^ and RNF138^KO^ HCT116 (left) and RKO (right) cells. Histogram shows the quantification of nuclear p65 intensity (*n* = 20). Scale bar, 5 μm. **m** qPCR analysis of NF-кB target genes expression in RNF138^WT^ and RNF138^KO^ HCT116 (top) and RKO (bottom) cells (*n* = 3). **P < 0.01 and ***P < 0.001

We constructed the tumor organoids system to understand RNF138’s role in the CRC tumorigenesis and progression (Fig. 3a), in which the growth of organoids was monitored for 9 days from day 0.5 post transplantation. From day 7, larger in diameter became apparent in the organoids derived from the *RNF138*^−/−^ tumors (Fig. 3b; Supplementary Fig. S4a) along with alternated morphology and more rapid increase in number (Fig. 3c; Supplementary Fig. S4b). The xenografts from the *RNF138*^−/−^ organoids developed larger in volume, on average two times of the ones from the control (Fig. 3d; Supplementary Fig. S4c, d), and exhibited a higher level of excessive growth indicated by Ki67-staining (Fig. 3e). Altogether, these results confirmed that RNF138 suppresses the tumor growth independent of the orthotopic microenvironment.

### RNF138 dysregulation gives rise to aberrant NF-кB signaling underlying chronic inflammation, colonic neoplastic transformation, and progression

We systematically examined the global changes imposed by *RNF138* deficiency in either the colon tissues dissected from the colitis and CAC models or the organoids derived from the CAC model. In all three sets of samples, substantial differences were identified at transcriptomic level between the *RNF138*^fl/fl^ and *RNF138*^−/−^ genetic backgrounds (Fig. 3f; Supplementary Fig. S5a). As revealed in the gene ontology (GO) term analysis, these differences primarily attributed to changes in the intestinal epithelial cell differentiation, proliferation, apoptosis, and cellular senescence (Supplementary Fig. S5b). Further analysis of signaling networks underlying these functional changes illuminated the central role of the NF-κB signaling interconnecting with the diverse functional elements (Fig. 3g, h; Supplementary Fig. S5c, d). Nuclear staining of p65 as an indicator downstream of NF-κB signaling were significantly enhanced upon *RNF138* deletion (Fig. 3i, j). Thus, RNF138 is functionally linked to the NF-κB signaling that is critical for a cascade of events from the onset of colitis to the colitis-to-tumor transition and further CAC tumor progression.

We also recapitulated this phenomenon in human CRC cell lines where the *RNF138* expression was suppressed *via* specific small interfering RNA (siRNA). In both HCT116 and RKO cells, upon *RNF138* RNA*i*, p65 levels were moderately increased though with significant increase in the phosphorylation of p65 (pp65) (Supplementary Fig. S5e), consistent with results observed when disrupting RNF138 by CRISPR-Cas9 (Fig. 3k). Furthermore, RNF138 deficiency enhanced the nuclear translocation of p65 (Fig. 3l) that led to the increase in the downstream gene expression (*NFκB1*, *IκBα*, *CXCL1*, and *IL8*), overall highlighting enhanced activation of the NF-κB signaling pathway (Fig. 3m; Supplementary Fig. S5f). In brief, RNF138 negatively regulates the NF-κB signaling during colorectal tumorigenesis.

### RNF138 restrains the activation of NF-κB signaling by retaining NIBP in the nucleus

**Fig. 4:**
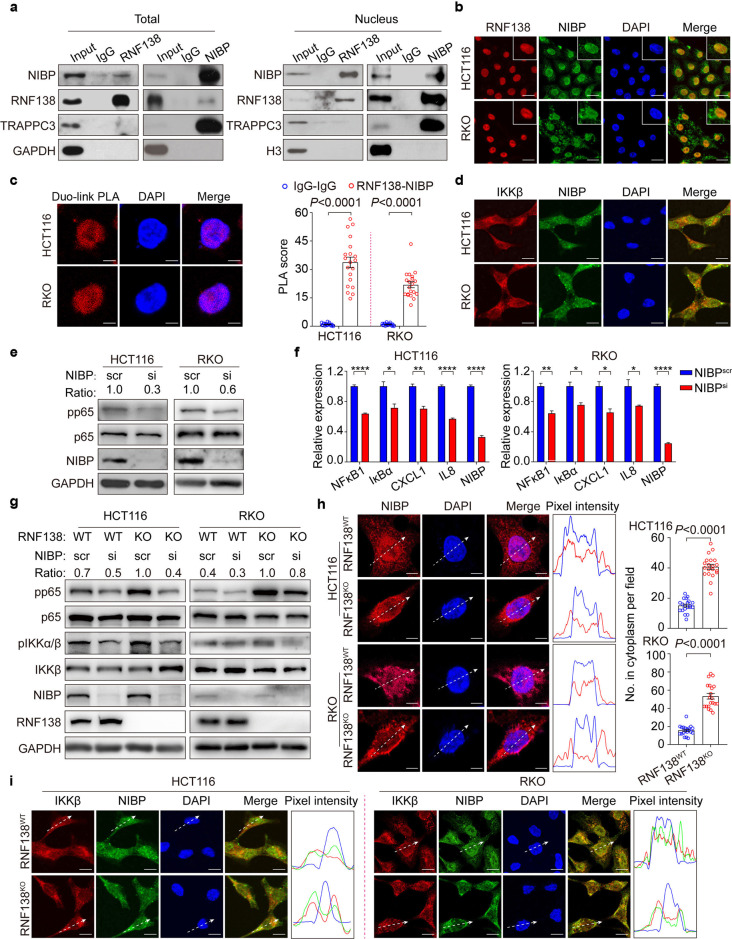
Nuclear-cytoplasmic partitioning of NIBP is regulated by RNF138 and contributes to the activation of NF-κB signaling. **a** Co-immunoprecipitation analysis of endogenous RNF138 and NIBP interactions in HCT116 total lysates (left) and nuclear extracts (right). IgG was used as control. **b** Immunostaining analysis of RNF138-NIBP co-localization in HCT116 and RKO cells. Nuclei were stained with DAPI. Insets show stained areas in more detail. Scale bar, 20 μm. **c** Representative confocal images (left) and quantification (right, *n* = 20) of endogenous RNF138 and NIBP interactions in HCT116 and RKO cells using proximity ligation assay. IgG were used as controls. Scale bar, 5 μm. **d** Immunostaining analysis of the co-localization of endogenous IKKβ and NIBP in HCT116 and RKO cells. Scale bar, 5 μm. **e** Immunoblots of phosphorylation, total levels of p65 in HCT116 and RKO cells transfected with si-NIBP or control siRNA. **f** qPCR analysis of NF-κB target gene expression in HCT116 and RKO cells (*n* = 3) with NIBP inhibition. *P < 0.05; **P < 0.01 and ****P < 0.0001. **g** Immunoblots of phosphorylation and total levels of p65 and IKKβ in RNF138^WT^ and RNF138^KO^ HCT116 and RKO cells with NIBP inhibition. **h** Representative immunostaining images of NIBP localization in RNF138^WT^ and RNF138^KO^ HCT116 and RKO cells, and the distribution of relative fluorescence intensities of lines scanned across the nucleus (right). Quantification of cytoplasmic NIBP intensity in HCT116 and RKO cells (*n* = 20). Scale bar, 5 μm. **i** Immunostaining analysis of IKKβ-NIBP co-localization in RNF138^WT^ and RNF138^KO^ HCT116 and RKO cells and relative pixel intensity of lines scanned across the nucleus. Scale bar, 20 μm

RNF138 seems not engaged in the canonical NF-κB signaling pathway directly (Supplementary Table 5; Supplementary Fig. S6a) but through the association with NIK- and IKK-β-binding protein (NIBP), a NF-κB signaling transduction regulator identified previously.^30,31^ The interaction between RNF138 and NIBP was confirmed by immunoprecipitation, and most likely occurs in the nucleus (Fig. 4a) as NIBP distributes in both the nucleus and cytoplasm whereas RNF138 resides primarily in the nucleus (Fig. 4b). As revealed in the proximity ligation assay, NIBP interacts with RNF138 directly in the nucleus (Fig. 4c) whereas it is associated with IKKβ (Fig. 4d), in the cytoplasm.^30^ Upon the RNA*i* against NIBP, the activation of NF-κB was significantly compromised, which was reflected in the reduction both in the phosphorylation of p65, (Fig. 4e) and in the transcription of downstream genes featured by *NFкB1, IκBα, CXCL1, and IL8* (Fig. 4f), suggesting that NIBP likely mediates the regulation of NF-κB by RNF138. Furthermore, the loss of *NIBP* restored the NF-кB signaling that was distorted due to the defective RNF138 (Fig. 4g). In summary, RNF138 regulates the NF-κB signaling at least partially via NIBP.

RNF138 serves as a Ub-E3 ligase in DNA damage response.^22,23,32,33^ In contrast, the level of NIBP remained unchanged upon the disruption of RNF138 by either RNA*i* or knockout (Supplementary Fig. S6b), suggesting the functional association between two is likely independent of ubiquitination and protein-degradation. Interestingly, the paradigm of NIBP subcellular distribution was shifted upon *RNF138* depletion, with significant increase in the nuclear partitioning coincided with the decreased cytoplasmic presence (Fig. 4h), which consequently enhanced the co-localization of NIBP and IKKβ as observed in the RNF138-deficient HCT116 and RKO cells (Fig. 4i).

Thus, we propose that the nuclear-cytoplasmic partitioning of NIBP contributes significantly to the key mechanism by which RNF138 regulates the NF-κB signaling transduction.

### The Ub-E3 ligase activity is dispensable for the regulation of NIBP by RNF138

**Fig. 5:**
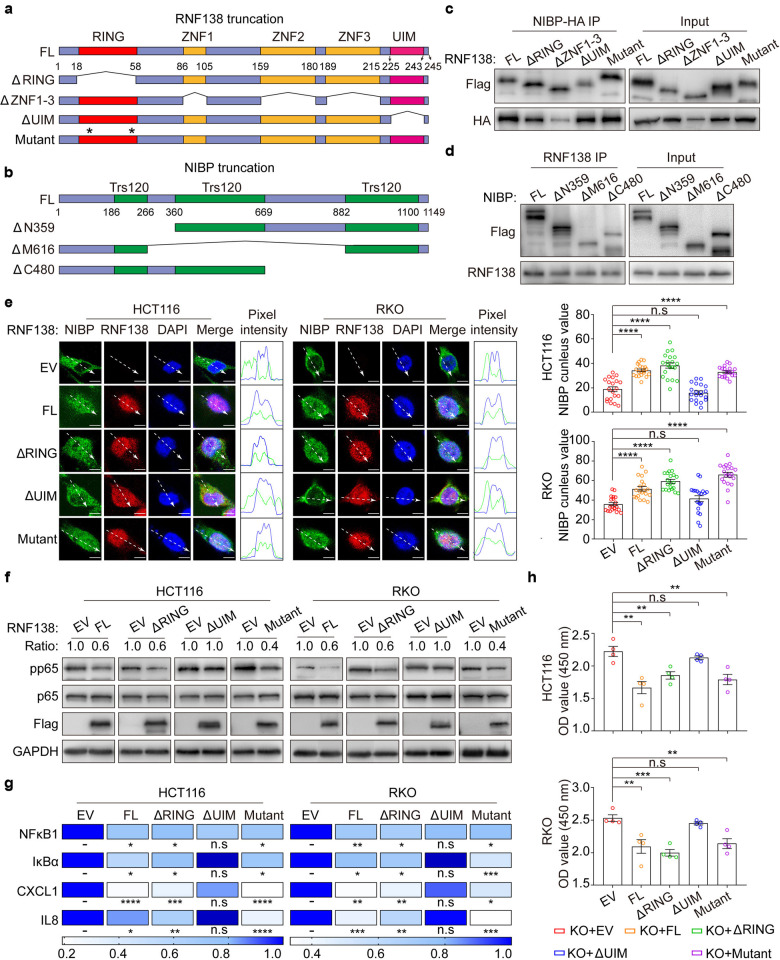
The regulation of NIBP by RNF138 is independent of the Ub-E3 ligase activity. **a** Schematic representation of RNF138 mutant constructs. Asterisk indicates amino acid residue mutant location (C/A) in RING domain. **b** Schematic representation of NIBP truncation constructs map. **c** Co-IP analysis of the interaction between HA-NIBP and full-length or truncated Flag-RNF138 mutants in 293T cells. **d** Co-IP analysis of the interaction between RNF138 and Flag-NIBP truncations in 293T cells. **e** Representative images of NIBP-immunostaining in HCT116 and RKO RNF138^KO^ cell lines transfected with empty vector or RNF138 truncations (flag-red), with co-localization as shown by the pixel intensity profile. Quantification of NIBP nucleus intensity in HCT116 and RKO cells transfected with different RNF138 mutants (right). Scale bar, 5 μm. **f** Immunoblots of phosphorylation and total p65 levels in HCT116 and RKO-knockout cells transfected with EV or different RNF138 truncations. **g** qPCR analysis of NF-κB target gene expression in HCT116 and RKO-knockout cells transfected with EV or RNF138 truncations. **h** Cell viability of HCT116- and RKO-knockout cells transfected with EV or RNF138 truncations

The ubiquitin interacting motif (UIM) located in RNF138 extreme C-terminus (AA225-243) is essential for its interaction with NIBP as opposed to the RING domain (AA18-58) and triple Zinc finger (ZNF) (ZNF1 AA86-105, ZNF2 AA159-180, and ZNF3 AA189-215) (Fig. 5a, c). However, little impact on the interaction between RNF138 and NIBP was observed when RNF138, as a Ub-E3 ligase, became inactive with specific mutations introduced to the catalytic cysteines (C18A/C54A) (Fig. 5c). We also identified the second (AA360-669) and the third Trs120 domain (AA882-1100) in NIBP were primarily responsible for its interaction with RNF138 (Fig. 5b, d).

This molecular connection was also reflected at functional level. The distorted nuclear partitioning of NIBP due to *RNF138* deficiency could be restored by ectopically expressing either the full-length protein (FL), RING domain protein-truncated protein (ΔRING) or the catalytic cysteines C/A mutant (Mutant), but much less effective with the UIM-truncated protein (Fig. 5e). Furthermore, in contrast to the UIM-truncated protein, either the full length, the RING domain-truncated, or the catalytic cystine C/A mutant could restore the NF-κB signaling distorted by the loss of RNF138, which was manifested first by changes in the phosphorylation of p65 (Fig. 5f), then the expression of downstream genes including *NF-кB1*, *IκBα*, *CXCL1*, and *IL8* (Fig. 5g), and further in cell proliferation (Fig. 5h). Importantly, the UIM-truncated RNF138 failed to exert impact on the proliferation as opposed to the full-length protein, the RING domain-truncated, and the catalytic cystine C/A mutant RNF138. Therefore, RNF138 regulates NF-κB signaling through its physical and functional interaction with NIBP independent of its Ub-E3 ligase activity.

### *RNF138* downregulation coincides with the aberrant NF-кB signaling in CRC associated with unfavorable clinical outcomes

**Fig. 6:**
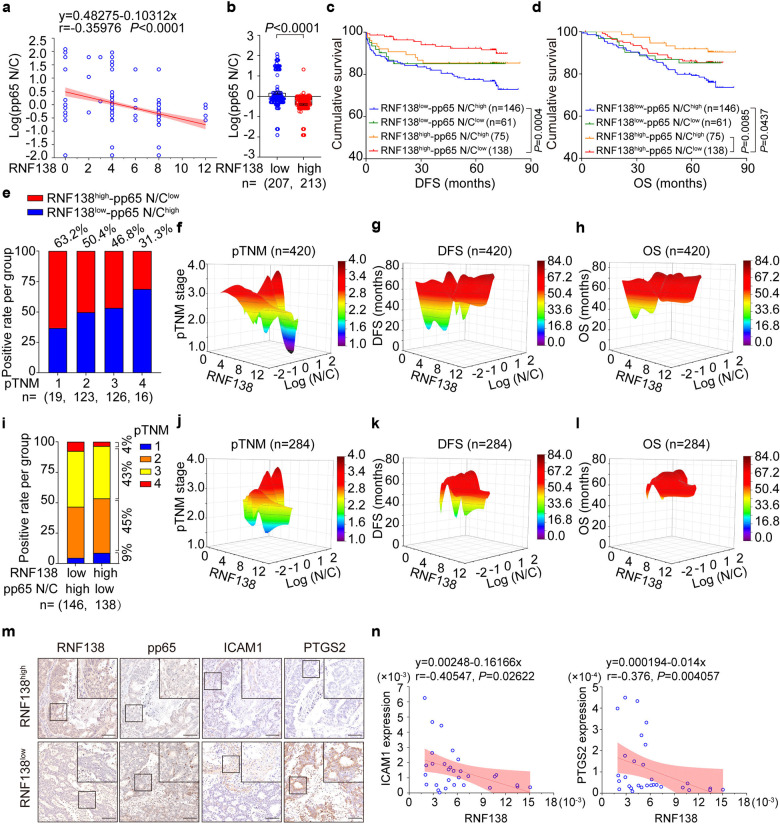
*RNF138* reduction coincided with aberrant activation of NF-кB signaling and correlated with unfavorable clinical outcomes in the CRC patients. **a** Correlation between log(pp65 N/C) and RNF138-staining according to Pearson’s test. The formula, coefficient of correlation (r), and the *P* value are indicated (*n* = 420). **b** Assessment of log(pp65 N/C) expression levels in low (*n* = 207) and high (*n* = 213) RNF138 CRC tissues. Data are mean ± SEM. Kaplan–Meier survival curves of DFS (**c**) and OS (**d**) stratified by RNF138 and pp65 N/C expression in CRC TMAs (*n* = 420). Log-rank test was used. **e** Percentages of RNF138^high^-pp65 N/C ratio^low^ (red) and RNF138^low^-pp65 N/C ratio^high^ (blue) according to pTNM stage (1, 2, 3, 4) (*n* = 284). Correlation among RNF138, log(pp65 N/C), and pTNM stage (**f**), DFS (**g**), and OS (**h**) in CRC patient TMAs (*n* = 420). Log(N/C) indicates log(pp65 N/C). **i** Percentages of pTNM stage in RNF138^high^-pp65 N/C ratio^low^ (*n* = 138) and RNF138^low^-pp65 N/C ratio^high^ (n = 146) in CRC patient TMAs (*n* = 284). Correlation among RNF138, log(pp65 N/C), and pTNM stage (**j**), DFS (**k**), and OS (**l**) in CRC patient TMAs (*n* = 284). Log(N/C), log(pp65 N/C). **m** IHC analysis of pp65, ICAM1, and PTGS2 in low- and high-RNF138-expressing CRC samples. Scale bar, 100 μm. **n** Correlation between RNF138 mRNA levels and NF-κB target genes according to qPCR analysis. The formula, coefficient of correlation (r), and the *P* value were provided *via* Pearson’s test (*n* = 30)

A significant positive correlation was established between RNF138 expression and p65 total level using human primary CRC TMAs (n = 420) (Supplementary Fig. S7a). Similar association was also established between RNF138 expression with the cytoplasmic accumulation of p65 as opposed to the nuclear translocation of p65 as an indicator of NF-кB activation in CRC tissue specimens (Supplementary Fig. S7b, c), suggesting that RNF138 negatively regulates the NF-кB signaling transduction. We further observed significant downregulation of RNF138 in the CRC tissue specimens that coincided with the hyperactivation of NF-кB signaling pathway indicated by the changes in the p65 nucleic/cytoplasmic (N/C) ratio (Fig. 6a, b). Specifically, the CRC patients were stratified into four groups based on the level of RNF138 and the pp65 N/C ratio, i.e., RNF138^high^-pp65 N/C ratio^low^, RNF138^low^-pp65 N/C ratio^high^, RNF138^high^-pp65 N/C ratio^high^, and RNF138^low^-pp65 N/C ratio^low^. The Kaplan–Meier survival analysis shown that CRC patients with RNF138^low^-pp65 N/C ratio^high^ had shorter disease-free survival and overall survival compared with the other three CRC subtypes (Fig. 6c, d). Importantly, RNF138^low^-pp65 N/C ratio^high^ tumors exhibited much more aggressive clinical features (Fig. 6e, f; Supplementary Fig. S7d, e). Moreover, RNF138, the pp65 ratio (N/C), and the progression and prognosis of CRC patients were inversely correlated (Fig. 6g, h; Supplementary Fig. S7f). In particular, the sustained activation of NF-кB signaling occurred in two RNF138-ablated CRC subtypes associated with much more aggressive tumors and adverse outcomes, indicating the inverse correlation between RNF138 expression and the pp65 N/C ratio in tumors (*n* = 284) (Fig. 6i–l; Supplementary Fig. S7g, h), consistent with the changes in downstream gene expression highlighted in the cases of ICAM1 and PTGS2 (Fig. 6m, n).

In brief, poor prognosis in CRC patients significantly correlates with the elevated NF-кB signaling coincided with *RNF138* downregulation in the affiliated tumors. Therefore, we highlighted the possibility of using RNF138 downregulation as indicator for both prognosis and the therapeutic interventions targeting the NF-κB signaling.

### Targeting the NF-кB signaling suppresses CRC growth associated with RNF138-ablation

**Fig. 7:**
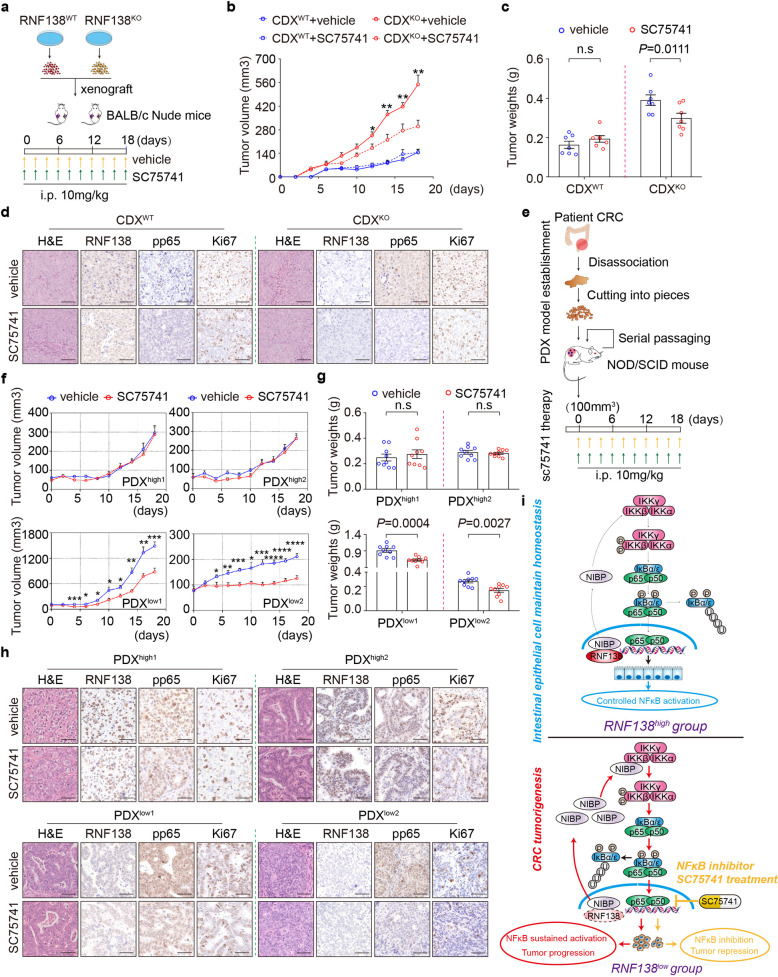
Targeting NF-кB signaling suppresses the RNF138-ablated CRC tumor growth. **a** Schematic overview of HCT116 CDX model establishment and SC75741 treatment. **b** The tumor growth curve of CDX model in RNF138^WT^ and RNF138^KO^ HCT116 cells treated with vehicle or SC75741 for 18 days *n* = 7. **c** Quantification of tumor weights from CDX model 18 days post-implantation (*n* = 7). **d** Representative H&E and IHC-staining of RNF138, pp65, and Ki67 from nude mice tumors. Scale bar, 25 μm. **e** Schematic overview of PDX model establishment and SC75741 treatment. **f** Tumor growth curve of PDX model of RNF138^high^-pp65 N/C ratio^low^ and RNF138^low^-pp65 N/C ratio^high^ treated with vehicle or SC75741 for 18 days (*n* = 9). **g** Quantification of tumor weights from PDX models on the 18th day post-injection SC75741 (*n* = 9). **h** Representative H&E and IHC-staining with anti-RNF138, -pp65, and -Ki67 antibodies in tumor tissue from PDX models. Scale bar, 25 μm. **i** Proposed model for the role of NF-κB signaling regulated by RNF138 in colonic neoplastic transformation, progression, and SC75741 treatment

We then further explored the therapeutic potential in targeting the distorted NF-κB signaling associated with RNF138 downregulation. SC75741 appeared effective in suppressing CRC tumor cell growth by the NF-κB pathway (Supplementary Fig. S8a–d), and could restore the aberrant NF-κB signaling with deficient RNF138-NIBP axis (Supplementary Fig. S8e). We therefore selected SC75741 as a specific chemical probe to address the effect of targeting the NF-κB signaling on the CRC progression. The *RNF138*^*WT*^ and *RNF138*^*KO*^ xenografts (CDX) were generated in parallel by injecting the corresponding HCT116 cells into the nude mice prior to the SC75741 treatment (Fig. 7a). Upon the treatment, the tumor growth was remarkably repressed in the *RNF138*^*KO*^ xenografts, reflected in the reduction in both tumor volume and weights, whereas no significant difference was observed in the *RNF138*^*WT*^ counterparts (Fig. 7b, c; Supplementary Fig. S9a, b). This inhibition was also marked at molecular level by the significant reduction in the Ki67 and PCNA staining of the *RNF138*^*KO*^ xenografts compared with the WT, indicating an impaired proliferation as the result of the inhibition of NF-κB signaling (Fig. 7d; Supplementary Fig. S9c).

We further evaluate the results in a more clinical-relevant context with the CRC patient-derived xenograft (PDX) models (Fig. 7e). Specifically, the corresponding fragments derived from either RNF138^high^-pp65 N/C ratio^low^ (patient-1 and patient-2) or RNF138^low^-pp65 N/C ratio^high^ (patient-3 and patient-4) CRC tumors were transplanted into NOD/SCID mice for PDX models (Supplementary Fig. S9f; Supplementary Table 6). SC75741 effectively blocked the activation of NF-κB signaling in all these models and specifically inhibited the growth of the RNF138^low^-pp65 N/C ratio^high^ PDX model (Fig. 7f, g; Supplementary Fig. S9d, e). In contrast, no significant change was observed in the RNF138^high^-pp65 N/C ratio^low^ PDX models (Fig. 7f, g; Supplementary Fig. S9e). The Ki67- and PCNA-staining also indicated specific reduction in cell proliferation, accompanied by the diminished NF-κB signaling, in the RNF138^low^-pp65 N/C ratio^high^ model (Fig. 7h; Supplementary Fig. S9g).

Collectively, these results strongly support the role of aberrant activation of NF-κB pathway in the CRC progression that is specially associated with *RNF138* dysregulation. We propose NF-κB signaling-targeted therapy as the potential effective clinical intervention for the CRC with poor prognosis specifically associated with RNF138 downregulation (Fig. 7i).

## Discussion

RNF138 functions as a Ub-E3 ligase to protect genome stability.^22,23,34^ However, mounting evidence suggests more complex functionality around RNF138, particularly between its *RNF138* expression with carcinogenesis and chemoresistance.^35,36^ Multiple studies confirmed differential expressions of RNF138 in different physiological and pathological settings.^36–38^ Therefore, it is conceivable that RNF138 may reveal different aspects of its functionality under different circumstances. For example, RNF138 drives NF-κB activation and lymphomagenesis through destabilizing MYD88 L265P by ubiquitination,^25^ which differs from the mechanism proposed by this work. We revealed here a close association between *RNF138* expression and NF-κB-mediated inflammation that is central to CRC tumorigenesis and prognosis. Using the ex vivo colitis and CAC models, we confirmed that RNF138 functions as a suppressor of CRC tumorigenesis through preventing excessive activation of NF-κB signaling. These findings together raise a possible interplay between DNA damage and repair process and inflammation in the context of tumorigenesis, particularly when considering the increasing amount of evidence showing that disrupting DNA damage response and repair accelerates inflammation.^39,40^ On the other hand, loss of cell/tissue integrity as well as other pathological alterations due to chronic inflammation leave higher vulnerability of DNA damages and potential mutations as the base of tumorigenesis.^41,42^ Hence investigating the role of RNF138 in these medical contexts could provide crucial insights into the interplay between these two processes. However, no significant correlation was observed between RNF138 and DNA damage response, indicated by γH2AX, in our colitis, CAC, or CAC-derived organoids models (Supplementary Fig. S10a, b), therefore in line with the notion of complex functionality of RNF138 in different physiological and pathological settings. RNA-seq analysis of *RNF138*-deficient models and in-depth data analysis revealed NF-кB signaling as the key target of RNF138 whereas other CRC-correlated genetic pathways, such as Wnt signaling and STAT3 signaling, were not remarkably altered with RNF138 depletion (Supplementary Fig. S10c–f). In the same line, the enhanced activation of NF-кB signaling occurred in CRC cells with RNF138 deficiency, implying a new regulatory mechanism for NF-кB signaling by RNF138. Although we appreciate the fact that RNF138’s E3 ligase activity may play important roles in different tumorigenesis processes,^25^ our work nevertheless highlights its functionality aspects independent of the E3 ligase activity by showing its control of the nucleocytoplasmic partition of NIBP as a key regulator of NF-κB signaling. Specifically, the UIM domain of RNF138 mediates its interaction with NIBP, critical for balancing the activation of NF-κB.

In diverse cancer types, the regulation of NF-кB signaling frequently become distorted, resulting in constitutive and unwarranted activation of the pathway associated with chronic inflammation.^12,15^ Therefore, reversing this process by targeting the NF-кB signaling have been an attractive direction in therapeutic development.^43^ Significant advance in the field is manifested in a collection of promising NF-кB inhibitors both in the development pipeline and in clinic.^44–46^ However, much left behind is our knowledge on the prerequisites for determining the timing and potential clinical outcome of such interventions. Particularly, effective early clinical indicators of aberrant NF-кB pathways are lacking, greatly hindering the applications of corresponding chemotherapeutic innovations. We demonstrated that downregulation of *RNF138* significantly promoted the transition from colitis to tumor and sensitized the CRC cells to SC75741, a highly potent and specific NF-κB signaling inhibitor. More importantly, this notion was further supported by the observations in the CDX and PDX models, highlighting the potential of SC75741 in specific NF-κB-targeting therapy for treating the CRC patients with low *RNF138* expression and hyperactivation of NF-κB (high pp65 N/C ratio). Critically, this clinical index was associated with a significant proportion (~34.8%, [146/420]) of CRC patients in our cohort, and with poorer prognoses compared with the rest of the cohort. This prompts us to wonder the possibility that low RNF138 and high pp65 N/C ratio in combination can be utilized as a useful indicator in clinic for the choice of NF-κB chemotherapeutic strategies.

In conclusion, our work promotes the potential of targeting NF-κB signaling in chemotherapy for CRC by dissecting the molecular mechanism underlines the aberrant activation of NF-κB signaling coincided with the loss of RNF138 and addresses the importance of RNF138 as a prognosis indicator for such treatments (Supplementary Fig. S11).

## Materials and methods

### Human specimens

The primary CRC tissues and 420 CRC tissue microarrays (TMAs) with follow-up were obtained from the Cancer Hospital, Chinese Academy of Medical Sciences (Beijing, China). Patient consent and approval were obtained from the Institutional Research Ethics Committee for research purpose (NCC2019C-016). The 134 paired CRC specimens were obtained from Shanghai Biochip. The detailed clinicopathological characteristics of the patients are summarized in Supplementary Tables 3, 4 and 6.

### Mice

RNF138-deficient mice were generated as previously described.^24^ Female BALB/c nude mice and NOD/SCID mice were obtained from Beijing Vital River Laboratory Animal Technology. All protocols were approved by the Institutional Animal Care and Use Committee at the Institution of Basic Medical Sciences (005-2016).

### Cell culture

HCT116, RKO, and HEK293T cell lines were obtained from the Cell Resource Center of Peking Union Medical College (Beijing, China) and cultured as described elsewhere.^47^ All cell lines were maintained at 37 °C, in a 5% CO_2_ incubator and used before reaching 20 passages.

### Quantification and statistical analysis

A comparison of two groups was analyzed by Student’s *t*-test. Kaplan–Meier curves for survival were generated and analyzed by a log-rank test. The clinicopathological parameters in CRC cases were analyzed using Pearson’s chi-square test or Fisher’s exact chi-square test. Correlation studies were analyzed with Pearson’s or Spearman’s rank correlation tests. *P* value less than 0.05 were considered statistically significant. GraphPad Prism 8.0, SPSS 20.0, and Origin2019b were used to generate graphs and statistical analyses. The results are repeated at least three times and are presented as the means ± SEM for all quantified data, unless stated otherwise.

## Supplementary information

Supplementary Materials

## Data Availability

All data supporting the findings of this study are available from the corresponding authors on reasonable request.
