# Supplementary material for: RING finger 138 deregulation distorts NF-кB signaling and facilities colitis switch to aggressive malignancy
Source: Signal Transduct Target Ther. 2022 Jun 13;7:185. doi: 10.1038/s41392-022-00985-1 (PMC9192753; doi:10.1038/s41392-022-00985-1)
Supplement: Supplementary file 1 — Supplementary Materials [file 41392_2022_985_MOESM1_ESM.docx]

Supplementary Materials for

**RING finger 138 deregulation distorts NF-кB signaling and facilities colitis switch to aggressive malignancy**

Yalan Lu^1,2,3,4#^, Rong Huang^1,5#^, Jianming Ying^3^**^,^**^6#^, Xingchen Li^2,3#^, Tao Jiao^1^, Lei Guo^6^, Haitao Zhou^2,3^, Han Wang^1^, Amannisa Tuersuntuoheti^1^, Jianmei Liu^2,3^, Qichen Chen^2,3^, Yanhong Wang^1^, Luying Su^1^, Changyuan Guo^6^, Fu Xu^1^, Ziyi Wang^1^, Yan Lu^1^, Kai Li^1^, Junbo Liang^1^, Zhen Huang^2,3^, Xiao Chen^2,3^, Jinjie Yao^2,3^, Hanjie Hu^2,3^, Xiaowen Cheng^7^, Yufeng Wan^7^, Xinyan Chen^7^, Ning Zhang^8^, Shiying Miao^1^, Jianqiang Cai^2,3^, Linfang Wang^1^, Changzheng Liu^1*^, Wei Song^1*^, Hong Zhao^2,3*^

**Affiliations:**

1. Department of Biochemistry and Molecular Biology, State Key Laboratory of Medical Molecular Biology, Institute of Basic Medical Sciences Chinese Academy of Medical Sciences, School of Basic Medicine Peking Union Medical College; Beijing, 100005, China.

2. Department of Hepatobiliary Surgery, State Key Laboratory of Molecular Oncology, National Cancer Center/National Clinical Research Center for Cancer/Cancer Hospital, Chinese Academy of Medical Sciences and Peking Union Medical College; Beijing, 100021, China.

3. Key Laboratory of Gene Editing Screening and R＆D of Digestive System Tumor Drugs, Chinese Academy of Medical Sciences and Peking Union Medical College; Beijing, 100021, China.

4. Key Laboratory of Human Disease Comparative Medicine, Chinese Ministry of Health, Beijing Key Laboratory for Animal Models of Emerging and Remerging Infectious Diseases, Institute of Laboratory Animal Science, Chinese Academy of Medical Sciences and Comparative Medicine Center, Peking Union Medical College; Beijing, 100021, China.

5. National Cancer Center/National Clinical Research Center for Cancer/ Cancer Hospital & Shenzhen Hospital, Chinese Academy of Medical Sciences and Peking Union Medical College; Shenzhen, 518116, China.

6. Department of Pathology, State Key Laboratory of Molecular Oncology, National Cancer Center/National Clinical Research Center for Cancer/Cancer Hospital, Chinese Academy of Medical Sciences and Peking Union Medical College; Beijing, 100021, China.

7. Department of Clinical Laboratory, the First Affiliated Hospital, Anhui Medical University; Hefei, 230022, China.

8. Wellcome Centre for Anti-Infectives Research (WCAIR), Division of Biological Chemistry and Drug Discovery, School of Life Sciences, University of Dundee; Dundee, DD1 5EH, UK.

# Yalan Lu, Rong Huang, Jianming Ying, and Xingchen Li contributed equally to this work.

*Correspondence: Changzheng Liu (cz-liu@ibms.pumc.edu.cn) or Wei Song (songwei@ibms.pumc.edu.cn) or Hong Zhao (zhaohong@cicams.ac.cn)

**This PDF file includes:**

Materials and Methods

Figures S1 to S11

Tables S1 to S7

**Materials and Methods**

**Public database analysis**

The expression of RNF138 in 10 cancer types was based upon data generated by the TCGA Research Network (<http://cancergenome.nih.gov/>). TCGA disease codes and abbreviations: CRC, colorectal adenocarcinoma (combining COAD and READ projects); SKCM, cutaneous melanoma; LIHC, hepatocellular carcinoma; CHOL, cholangiocarcinoma, THCA, thyroid carcinoma; PAAD, pancreatic adenocarcinoma; HNSC, head and neck squamous cell carcinoma; THYM, thymoma; UCEC, uterine corpus endometrial carcinoma; LUAD, lung adenocarcinoma. *RNF138* expression was indicated as fragments per kilobase million (FPKM). Information on the expression of RNF138 in CRC was downloaded from the GEO database (https://www.ncbi.nlm.nih.gov/geo/) for accession numbers GDS4382, GDS2947, and GDS4718.

**RNF138 antibody**

An *RNF138* fragment containing amino acid residues 60-245 was inserted into the N-His-pET-28a expression vector. The construct was transformed into Transetta (DE3) [competent](C:/Users/lulu/AppData/Local/youdao/dict/Application/8.9.6.0/resultui/html/index.html#/javascript:;) [cell](C:/Users/lulu/AppData/Local/youdao/dict/Application/8.9.6.0/resultui/html/index.html#/javascript:;)s (TransGen Biotech, CD801-02, Beijing, China) and RNF138 fragment expression was induced by IPTG (0.8 mM，Sigma-Aldrich, I6758, MO, USA). The protein was separated by 8% SDS-PAGE, and the target band was triturated and dissolved in 8 M urea at 37°C for 1 hour. The supernatant was incubated with renaturing solution (4 M Urea, 50 mM Tris, 0.5 mM EDTA, 50 mM NaCl, 0.4 M Argine, 5 mM DTT, 0.2% Tween 20, 1 mM oxidized glutathione, and 2 mM reduced glutathione) overnight, followed by concentrating in an Amicon Ultra (Millipore, UFC901096, MA, USA) at 13,500 *g* for 10 minutes. Five female 6 to 8-week-old rat were injected with concentrated RNF138 protein fragment. After one month, the splenocytes of the immunized rat were fused with myeloma cells. The hybridomas were selected by hypoxanthine, aminopterin and thymidine (HAT) (Sigma-Aldrich, H0262) and the positive single-cell colonies were selected through serial dilution and verified by [immunoblot](C:/Users/lulu/AppData/Local/youdao/dict/Application/8.7.0.0/resultui/html/index.html#/javascript:;)ting analysis. Mice ascites fluid were generated and collected by injecting the positive hybridomas into BALB/c mice. RNF138 antibodies were purified from ascetic fluid and added glycerinum for further storage and application. The hybridoma cell strain (named SW308-1, 9G12) had been preserved at China General Microbiological Culture Collection Center (CGMCC No.19676). The antibody has been submitted to the China National Intellectual Property Administration (202010452272.0).

**RNA extraction and quantitative real-time PCR**

Total RNA was extracted from tissues (human CRC tissue) and cells (HCT116 and RKO) using TRIzol reagent (Invitrogen, 15596018, MA, USA) according to the manufacturer’s instructions. Then, cDNA was synthesized from equal amounts of total RNA extracts using the Revert Aid First Strand cDNA Synthesis Kit (ThermoFisher Scientific, K1622, MA, USA). Quantitative real-time PCR (qPCR) analyses of target genes (in tissues, *RNF138*, *PTGS2*, *ICAM1*, and beta actin [*ACTB*]; in cell lines, *RNF138*, *CXCL1*, *NFκB1*, *IκBα*, *IL8*, and *ACTB*) were performed using the Power Up SYBR Green Master Mix (Applied Biosystems, A25742, CA, USA) on the CFX Connect Real-Time Detection System (Bio-Rad, Hercules, CA, USA). The relative expression was calculated by the 2(-ΔΔCt) method, and the values were normalized to *ACTB* for each sample. The correlations of *RNF138* with *ICAM1* and *PTGS2* expression in 30 CRC specimens were examined with the Pearson’s test. The primer sequences used for qPCR are listed in Supplementary Table 7.

**Immunoblotting analysis**

Tissues or cell pellets were lysed in SDS lysis buffer (50 mM Tris-HCl [pH 6.8], 2% SDS, and 10% glycerol) supplemented with protease inhibitor cocktail (Roche, 11836170001, Basel, Germany) and PhosSTOP (Roche, 4906837001). After boiling for 5 minutes, the supernatant was collected by 13,500 *g* for 10 minutes. Equal amounts of extract (20-50 μg) were quantified with a BCA protein assay kit (Pierce Biotechnology, 23225, IL, USA) and separated by 8-15% SDS-PAGE for 1.5-2 hours, followed by protein transfer onto polyvinylidene difluoride (PVDF) membranes (GE Healthcare Life Sciences, RPN303F, IL, USA). After blocking with 5% (w/v) bovine serum albumin (BSA) or skim milk, the membranes were incubated with primary antibody at 4°C overnight followed by incubation with the HRP-conjugated second antibody (Zhongshan Golden Bridge Biotechnology, PV-9001, PV-9002, PV-9004, Beijing, China) for 1 hour. The primary antibodies used were as follows: rat anti-RNF138 (1:1000 dilution, made in our lab), rabbit anti-NIBP (1:1000 dilution, Proteintech, 16014-1-AP, PA, USA), rabbit anti-TRAPPC3 (1:1000 dilution, Proteintech, 15555-1-AP), rabbit anti-phospho-NF-κB p65 (1:1000 dilution, Cell Signaling Technology, 3033, MA, USA), rabbit anti-NF-κB p65 (1:1000 dilution, Cell Signaling Technology, 8242), rabbit anti-phospho-IKKα/β (1:800 dilution, Cell Signaling Technology, 2697), rabbit anti-IKKβ (1:1000 dilution, Cell Signaling Technology, 8943), mouse anti-GAPDH (1:2000 dilution, Proteintech, 60004-1-Ig), mouse anti-Flag (1:1000 dilution, Sigma-Aldrich, F1804), and HA (1:1000 dilution, Cell Signaling Technology, 3724). Interesting protein bands were visualized with the ECL detection system (Millipore, WBULS0500). Quantification of the density of each indicator protein was normalized to the housekeeper protein with Image J software (NIH, USA).

**Immunostaining**

HCT116 and RKO cell lines were grown on glass coverslips (NEST, 801008, Jiangsu, China), fixed with 4% paraformaldehyde (PFA) solution (Servicebio, G1101, Wuhan, China) for 15 minutes, and permeabilized with 0.5% Triton-X 100 (Sigma-Aldrich, T8787) for 10 minutes. After blocking for non-specific binding with 10% (v/v) normal goat serum (Zhongshan Golden Bridge Biotechnology, ZLI-9021) for 30-40 minutes at room temperature, the cells were incubated with the indicated primary antibody at 4°C overnight. The primary antibodies were as follows: rat anti-RNF138 (1:50 dilution, made in our lab), rabbit anti-NF-κB p65 (1:50 dilution, Cell Signaling Technology, 8242), rabbit anti-NIBP (1:50 dilution, Proteintech, 16014-1-AP), rabbit anti-IKKβ (1:50 dilution, Cell Signaling Technology, 8943), mouse anti-Flag (1:100 dilution, Sigma-Aldrich, F1804). Then the cells were fluorescently probed with the secondary antibodies (1:500 dilution, Invitrogen, A32733, SA5-10168, A-11006) for 60 minutes. The slides were sealed with mounting medium containing 4′,6-diamidino-2-phenylindole (DAPI) (Zhongshan Golden Bridge Biotechnology, ZLI-9557), and immunofluorescence images were acquired under a confocal fluorescence microscope.

Both human and mouse CRC tissues were fixed with 4% PFA for 24 hours, cryoprotected in 30% sucrose-PBS, and embedded in Cry-Gel (Leica, 39475237, IL, USA). Cryosections of 20 μm thickness used for immunostaining analysis were washed in PBS (0.14 M NaCl, 0.0027 M KCl, 0.01 M Na_2_HPO_4_, and 0.002 M KH_2_PO_4_) and permeabilized with 0.5% Triton X-100 (Sigma-Aldrich, T8787), followed by blocking with 10% goat serum (Zhongshan Golden Bridge Biotechnology, ZLI-9021) and overnight incubation in primary antibody. The primary antibodies were as follows: rat anti-RNF138 (1:100 dilution, made in our lab), rabbit anti-Ki67 (1:100 dilution, Abcam, ab16667, Cambridge, UK), rabbit anti-NF-κB p65 (1:100 dilution, Cell Signaling Technology, 8242), rabbit anti-phospho-NF-κB p65 (1:100 dilution, Cell Signaling Technology, 3033), rabbit anti-γH2AX (1:100 dilution, Cell Signaling Technology, 9718), rabbit anti-β-Catenin (1:100 dilution, Cell Signaling Technology, 8480), mouse anti-STAT3 (1:100 dilution, Cell Signaling Technology, 9139). The sections were probed with AlexaFluor-conjugated secondary antibody (1:500 dilution, Invitrogen, A32733, SA5-10168, A-11006) for 2 hours at room temperature and sealed with mounting medium containing DAPI (Zhongshan Golden Bridge Biotechnology, ZLI-9557) for fluorescent signal analysis.

**TMA immunohistochemical analysis**

TMA immunohistochemical analysis was performed on routinely processed paraffin-embedded sections. Briefly, the TMAs were deparaffinized and rehydrated, followed treatment with antigen retrieval solutions for 10 minutes (RNF138: EDTA [pH 9.0], pp65: citrate [pH = 6.0]; Zhongshan Golden Bridge Biotechnology, ZLI-9069, ZLI-9064). We blocked endogenous peroxidase activity by incubating in 3% H_2_O_2_ for 10 minutes and non-specific binding by incubating in 10% goat serum for 1 hour at room temperature. The primary antibody (rat anti-RNF138 [1:100 dilution, made in our lab] or rabbit anti-pp65 [1:200 dilution, Abcam, ab86299]) was incubated and detected with the Polink-2 HRP Plus Polymer Detection System and DAB kit (Zhongshan Golden Bridge Biotechnology, [ZLI-901](https://www.so.com/link?m=a13eIrlqTBpQPR4pV4ID6UIhmTW1Qep71AUpgDgPs4Bmu32V63JZiTSEzvvJDBNyMeHJ9jOYzO16%2BnFYzvhMkwiXsEU4A%2Fmh8rp9W%2FOk7JScj4%2FEoJ51js6Ez8JUPRiTExv8DytVk0zhickllXZjm502PsO27WtxmUrnXBP9tiP8OdQHEWzcSK46trgMwpl%2FltvIEJOA9gBVsyyN38Rb%2F1NsPp1zDGb9ArVX0GuRK0NrhJQhCoD4lmtnWcc7FTbpiulpaMG51dEzQwHkpYNX7DIV%2Fhlo%3D)8) for standard immunohistochemical analysis. The arrays were scored independently by two pathologists for both the staining intensity and percentage of stained cells across the sections. The intensity score was assigned as 0, negative; 1, weak; 2, moderate; and 3, strong. The proportion score was assigned as follows, 0, 0%; 1, 1-24%; 2, 25-49%; 3, 50-74%; and 4, 75-100%. Each sample score was evaluated by the product staining intensity multiplied by the positive proportion, resulting overall scores ranging from 0 to 12. For pp65, both cytoplasmic and nuclear staining scores were assessed. The pp65 N/C ratio was the odds ratio of pp65 (nuclear score + 0.1) and (cytoplasmic score + 0.1). The optimal cutoff values for RNF138 (cutoff: 5) and pp65 (N/C) ratio (cutoff: 0.71) were calculated by ROC curve analysis based on the Youden index using SPSS 20.0 (IBM, USA). Images were observed under a light microscope.

**Chronic colitis and CAC model induction**

Male and female co-housed wild-type (RNF138^fl/fl^) and RNF138-deficient (RNF138^-/-^) mice were used for chronic colitis and CAC model induction at the age of 6 to 8 weeks. Chronic colitis was induced by oral administration of 2% (w/v) DSS (molecular weight: 36,000-50,000, MP Biomedicals, 215676080, CA, USA) in drinking water for 7 consecutive days, followed by a 14-day recovery period with normal water. The DSS solution was made fresh on 3th and 5th day of each cycle. This cycle was repeated twice, and mice were sacrificed 4 weeks after the last DSS cycle.

For the CAC model, all mice received a single intraperitoneal injection of AOM (Sigma-Aldrich, A5486) at a dose of 10 mg/kg body weight. After 4 days, 2% (w/v) DSS in drinking water was supplied over 7 days, followed by regular water for 2 weeks. This cycle was repeated twice, and mice were sacrificed 4 weeks after the last DSS treatment. Male and female mice maintained as chronic and CAC models and given no treatment were used as mock models.

At the endpoint of the chronic colitis, CAC, and mock model experiments, mice were sacrificed, their colons and spleens were harvested, and the lengths measured. Colons were then cut open longitudinally and washed with PBS. The gross tumor number was counted and sizes were measured. Tumor load was calculated according to the following formula: tumor load = (number of small tumors) × 1 + (number of medium tumors) × 2 + (number of large tumors) × 3. A portion of each tissue sample was fixed in 4% PFA and embedded in paraffin for histopathological analysis. The remaining tissues were snap-frozen in liquid nitrogen for RNA and protein analysis.

**Clinical assessment of colitis and CAC models**

During the chronic colitis and CAC study, mouse body weight, stool consistency, and hematochezia were routinely recorded every 3 days. Weight change was calculated as the percentage change compared with the baseline measurement. Stool consistency scores were determined as follows: 0, well-formed pellets; 1, semi-formed stools that did not adhere to the anus; 2, semi-formed stools that adhered to the anus; 3, liquid stools that adhered to the anus. Bleeding scores were determined as follows: 0, no blood; 1, positive hemoccult; 2, visible blood traces in stool; 3, gross rectal bleeding. The sum of stool consistency scores and bleeding scores were presented as clinical scores.^1^

**Histology and microscopy analyses**

Paraffin tissue sections (4-μm thick) from the chronic colitis and CAC models were deparaffinized, rehydrated, and stained with hematoxylin and eosin (H&E). Colitis scores were assigned based on the extent and severity of inﬂammation, ulceration, and hyperplasia of the mucosa. Scores for inflammation were as follows: 0, normal (within normal limits); 1, mild (small, focal, or widely separated, limited to lamina propria); 2, moderate (multifocal or locally extensive, extending to submucosa); 3, severe (transmural inflammation with ulcers covering >20 crypts). Scores for ulceration were as follows: 0, normal (no ulcers); 1, mild (1-2 ulcers involving up to a total of 20 crypts); 2, moderate (1-4 ulcers involving a total of 20-40 crypts); 3, severe (>4 ulcers or >40 crypts). Scores for hyperplasia were as follows: 0, normal (within normal limits); 1, mild (crypts 2-3 times normal thickness, normal epithelium); 2, moderate (crypts 2-3 times normal thickness, hyperchromatic epithelium, reduced goblet cells, scattered arborization); 3, severe (crypts >4 times normal thickness, marked hyperchromasia, few to no goblet cells, high mitotic index, frequent arborization). Scores for extent were as follows: 0, normal (0% involvement); 1, mild (up to 30% involvement); 2, moderate (30-70% involvement); 3, severe (>70% involvement).^1,2^ Differentiation grade was evaluated as high, moderate, or low. Histopathological evaluation was performed in a blinded fashion by at least two experienced pathologists. For the immunohistochemistry, paraffin-embedded tissue sections were stained with antibodies against Ki67 (1:200 dilution, Abcam, ab16667), NF-κB p65 (1:200 dilution, Cell Signaling Technology, 8242), β-Catenin (1:200 dilution, Cell Signaling Technology, 8480), STAT3 (1:100 dilution, Cell Signaling Technology, 9139), and γH2AX (1:100 dilution, Cell Signaling Technology, 9718).

**Isolation, culture, and xenotransplantation of tumor organoids**

Intestinal fragments containing tumors from CAC model mice were excised and incubated in EDTA chelation buffer (2 mM EDTA, 5.6 mM Na_2_HPO_4_, 8.0 mM KH_2_PO_4_, 96.2 mM NaCl, 1.6 mM KCl, 43.4 mM sucrose, 54.9 mM D-sorbitol, and 0.5 mM DTT in distilled water) for 60 minutes on ice to detach the normal epithelial cells. The attached tumor fragments were incubated in digestion buffer (2.5% FBS, 1 U/ml of penicillin, 1 μg/ml of streptomycin, and 2.5 ng/ml of amphotericin B, 200 U/ml type IV collagenase [GIBCO, 17104-019], 125 μg/ml type II dispase [GIBCO, 17105-041] in DMEM) for 2 hours at 37°C. Then the tumor fragments were settled under normal gravity for 1 minute, and the supernatant containing single tumor cells was collected. The washed tumor cells were resuspended in 5 mg/ml matrigel (BD Biosciences, 356237, NJ, USA) on ice and plated into 48-well plates at 1,000 cells per 25 μl of matrigel per well to polymerize for 15 minutes at 37°C. We then overlaid the matrigel with 250 μl/well basal culture medium (1 U/ml of penicillin, 1 μg/ml of streptomycin, and 2.5 ng/ml of amphotericin B, 10 mmol/L HEPES [ThermoFisher Scientific, [15630106](https://www.thermofisher.com/order/catalog/product/15630106)], 2 mM Glutamax [GIBCO, 35050-079], 1 × N2 supplement [Invitrogen, 17502-048], 1 × B27 supplement [Invitrogen, 17504-044], 1 mM N-acetylcysteine [Sigma-Aldrich, A9165-5G], and 50 ng/ml murine EGF [Invitrogen, 53003-018] in Advanced Dulbecco's Modified Eagle Medium/F12 [Invitrogen, 12634010]) for culture. ^3,4^

For xenotransplantation, tumor organoids cells were mechanically and enzymatically dissociated and resolved in ice-cold matrigel (Corning, 354234, NY, USA). Tumor cells at 5 ×10^5^ were subcutaneously injected into BALB/c nude mice via axilla skin. After 60 days, all mice were sacrificed, and the tumors were removed, photographed, and weighed. A portion of the tumors were fixed in 4% PFA for histological analysis, and the remaining tumors were snap frozen in liquid nitrogen for protein and RNA extraction.

**RNA-based next-generation sequencing**

Total RNA from chronic colitis-, CAC model-, and CAC-derived organoids were extracted using TRIzol (Invitrogen, 15596018) or RNeasy Mini Kit (Qiagen, 74104), followed by purification and analysis of the integrity and concentration of the cDNA libraries using the Agilent Bioanalyzer 2100 system. The libraries were sequenced on an Illumina Novaseq platform using 150-bp paired-end reads. Qualified sequencing reads were mapped to the reference mouse genome mm10. Aligned RNA-seq reads were assembled into transcripts using the Cufflinks program according to Gencode v.M8 (http://www.gencodegenes.org/). Samples were classified into different groups using principal component analysis (PCA). Differentially expressed genes were defined with thresholds of fold change ≥2 and padj <0.05. GO enrichment analysis of differentially expressed genes was implemented using the cluster Profiler R package, and the hypergeometric distribution was used to determine enrichment significance. [Gene Set Enrichment](https://www.sciencedirect.com/topics/biochemistry-genetics-and-molecular-biology/gene-set-enrichment) Analysis (GSEA) was applied to identify significantly changed pathways using the GSEA algorithm. Protein-protein interaction (PPI) analyses of interesting clusters and genes were based on the STRING database. The accession numbers for RNA sequencing data reported in this paper were uploaded to NCBI and deposited in GEO under accession number GSE144051.

**Small interfering RNA and plasmid transfections**

The RNF138 and NIBP siRNA oligonucleotides were designed and synthesized by the GenePharma company in Shanghai. The siRNAs were pooled to interfere with endogenous *RNF138* and *NIBP* expression. The interfering sequences are provided in Supplementary Table 7. Corresponding nonsense controls (scr) were carried out simultaneously. The siRNA transfection was performed using Lipofectamine RNAiMAX (Invitrogen, 13778150) at a final siRNA concentration of ~60 nM, according to the manufacturer’s instructions. After 24-48 hours, the cells were collected and analyzed by immunoblotting and qPCR.

**Generation of RNF138-knockout cells and stable cell lines**

To knockout cell expression with CRISPR genome-editing, an RNF138 genomic DNA sequence (AGGTGCTCAAAACGCCCGTG) was cloned into the lentiCRISP-v2-bsd vector (Addgene, 52961, MA, USA). The packaging plasmids psPAX2 (Addgene, 12260) and pMD2.G (Addgene, 12259) were co-transfected into HEK293T cells by Lipofectamine 2000 reagent (Invitrogen, 11668030). After 48 hours, the viral supernatants were harvested and used to infect HCT116 and RKO cells. Cells were selected by Blasticidin (HCT116: 2 µg/ml, RKO: 6 μg/ml) (Selleckchem, S7419, TX, USA) for 2 weeks. Single-cell colonies of each cell line were selected through serial dilution and verified by [immunoblot](C:/Users/lulu/AppData/Local/youdao/dict/Application/8.7.0.0/resultui/html/index.html#/javascript:;)ting analysis using the RNF138 antibody.

For stable knock-in cell lines, viruses were produced by transfecting HEK293T cells with pCDH-EF1-MCS-T2A-puro vectors (Addgene, 72263), carrying full-length and deletion/point mutants of human *RNF138* or the empty vector (EV), and the Lentiviral Packaging Mix (psPAX2 and pMD2.G). HCT116 and RKO knockout cells were infected with the viral supernatants followed by selection with puromycin (1.5 μg/ml, MP Biomedicals, 194539) for 1-2 weeks. Finally, the resistant homogenous knock-in clones with *RNF138* mutations were collected for further verification by immunoblotting analysis.

I**mmunohistochemistry**

Paraffin sections (4-µm thick) from mouse model and human tissues were heated at 60°C for 2 hours, deparaffinized by [xylene](C:/Users/lulu/AppData/Local/youdao/dict/Application/8.9.3.0/resultui/html/index.html#/javascript:;), and rehydrated with gradient ethanol immersion (100%, 100%, 95%, 80%, 75%, 50%) to PBS. Sections were then boiled in antigen-retrieval treatment with citrate (pH = 6.0) (for RNF138, EDTA [pH = 9.0]) for 10 minutes, followed by 3% H_2_O_2_ for 10 min at room temperature to quench endogenous peroxidase activity. Slides were blocked with 10% (v/v) goat serum for 30-40 minutes, and the sections were incubated with specific primary antibodies diluted in blocking solution at 4°C overnight. The primary antibodies were as follows: rat anti-RNF138 (1:200 dilution, made in our lab), rabbit anti-Ki67 (1:200 dilution, Abcam, ab16667), rabbit anti-NF-κB p65 (1:200 dilution, Cell Signaling Technology, 8242), rabbit anti-phospho-NF-κB p65 (1:200 dilution, Abcam, ab86299), rabbit anti-γH2AX (1:100 dilution, Cell Signaling Technology, 9718), rabbit anti-β-Catenin (1:200 dilution, Cell Signaling Technology, 8480), mouse anti-STAT3 (1:100 dilution, Cell Signaling Technology, 9139), rabbit anti-ICAM1 (1:100 dilution, Abcam, ab53013), and rabbit anti-PTGS2 (1:100 dilution, Cell Signaling Technology, 12282). After washing, the sections were incubated with Polink-2 HRP Plus Polymer Detection System according to the manufacturer’s instructions. The signals were visualized with the DAB kit (Zhongshan Golden Bridge Biotechnology, [ZLI-901](https://www.so.com/link?m=a13eIrlqTBpQPR4pV4ID6UIhmTW1Qep71AUpgDgPs4Bmu32V63JZiTSEzvvJDBNyMeHJ9jOYzO16%2BnFYzvhMkwiXsEU4A%2Fmh8rp9W%2FOk7JScj4%2FEoJ51js6Ez8JUPRiTExv8DytVk0zhickllXZjm502PsO27WtxmUrnXBP9tiP8OdQHEWzcSK46trgMwpl%2FltvIEJOA9gBVsyyN38Rb%2F1NsPp1zDGb9ArVX0GuRK0NrhJQhCoD4lmtnWcc7FTbpiulpaMG51dEzQwHkpYNX7DIV%2Fhlo%3D)8) and counterstained with hematoxylin.

**Plasmid construction**

The *RNF138* full-length (RNF138-FL) open reading frame was subcloned into the *Kpn* I and *Bam*H I sites of the p3 × Flag CMV14 vector (gift from Pro. Zhang Xiaodong Lab). A series of mutant p3 × Flag CMV14-RNF138 constructs were generated using the Quick-Change Site-Directed Mutagenesis kit (Stratagene, 200518, USA) for amino acid deletion or substitution according to manufacturer’s instructions. RNF138-ΔRING (amino acids: 1-17, 59-245), RNF138-ΔUIM (amino acids: 1-224, 244-245), RNF138-ΔZNF1 (amino acids: 1-85, 106-245), and RNF138-C18A were generated with p3 × Flag CMV14-RNF138-FL as the template. RNF138-ΔZNF1-2 (amino acids: 1-85, 106-158, 181-245) was generated with RNF138-ΔZNF1 as the template, and RNF138-ΔZNF1-3 (amino acids: 1-85, 106-158, 181-188, 216-245) was generated with RNF138-ΔZNF1-2 as the template. A RNF138-C/A double-mutant was generated with RNF138-C18A as the template.

The full-length of *NIBP* (NIBP-FL) was subcloned into p3 × Flag CMV14 and N-HA-pcDNA 6.0 vectors (gifts from Pro. Zhang Xiaodong Lab) between *Eco*R 1 and *Bam*H 1 sites. NIBP-ΔN359 (amino acids: 360-1149) and NIBP-ΔC480 (amino acids: 1-669) were subcloned into p3 × Flag CMV14. NIBP-ΔM616 (amino acids: 1-266, 882-1149) was generated by ligation between PCR products of NIBP-1-266 and NIBP-882-1149 and the p3 × Flag CMV14 vector. The primers used in PCR reactions are listed in Supplementary Table 7, and all plasmids were sequenced to confirm there were no errors.

**Co-immunoprecipitation (co-IP)**

For [endogenous](javascript:;) co-IP assays, HCT116 cell aggregates were lysed in RIPA buffer (20 mM Tris-HCl [pH 7.5], 150 mM NaCl, 1 mM Na_2_EDTA, 1 mM EGTA, 1% NP-40) (Cell Signaling Technology, 9806S) containing 1 × Complete EDTA-free protease inhibitor cocktail and 1 mM PMSF on ice. After centrifugation at 13,500 *g* for 15 minutes, the supernatants were incubated with the indicated antibodies (3 μg of RNF138, NIBP, or IKKβ) and the control rat (3 μg, Santa Cruz Biotech, sc-2026, TX, USA) or rabbit IgG (3 μg, Millipore, 12-370) antibody overnight at 4°C. After incubating with protein G (rat) or protein A (rabbit) agarose (Santa Cruz Biotech, sc-2002, sc-2001) at 4°C for 2 hours, the protein-antibody complexes were washed with the NETN buffer (20 mM Tris-HCl, pH 8.0, 100 mM NaCl, 0.5% NP-40, 1 mM EDTA) containing protease inhibitor cocktail. Complexes were eluted off the beads by boiling for 5 min in loading buffer, then subjected to immunoblotting analysis with antibodies against NIBP, RNF138, TRAPPC3, or GAPDH. An aliquot of each lysate was used as the input control.

For the exogenous co-IP assays, vectors encoding Flag-tagged (P3 × Flag CMV14-RNF138-wt/truncation/mt) and HA-tagged pcDNA6-HA-NIBP-wt proteins were co-transfected into HEK293T cells using lipofectamine 3000 (Invitrogen, L3000075) according to the manufacturer’s instructions. After 48 hours, cells were harvested and lysed in NETN buffer containing protease inhibitor cocktail. Cell extracts were incubated with anti-HA (3 μg, Cell Signaling Technology, 3724) overnight at 4°C, followed by incubating with DynabeadsTM Protein G (Invitrogen, 10004D) at 4°C for 3 hours. After washing three times, eluted proteins were boiled and subjected to immunoblotting analysis with antibodies against Flag (1:1000 dilution, Sigma-Aldrich, F1804) or HA (1:1000 dilution, Cell Signaling Technology, 3724). For the IP of flag-tagged NIBP, vectors encoding Flag-tagged (P3 × Flag CMV14-NIBP-wt/truncation) were transfected into HEK293T cell lines. After 48 hours, the cells were lysed in NETN buffer supplemented with protease inhibitor cocktail, and cell extracts were incubated with the RNF138 antibody, followed by incubation with Dynabeads protein G (30 µl, Invitrogen, 10004D). The protein-antibody complexes were washed with the NETN buffer and subjected to immunoblotting analysis with Flag and RNF138 antibodies.

**Nuclear fractionation**

Cell pellets were incubated in hypotonic buffer (10 mM HEPES [pH 7.9], 1.5 mM MgCl_2_, 10 mM KCl, 1 mM β-mercaptoethanol, 0.5 mM DTT, 0.5 mM PMSF) for 10-20 minutes on ice, and then flash-frozen in liquid nitrogen and stored at −80°C for at least 1 day. After thawing on ice, the cytoplasmic fraction (supernatant) was collected using centrifugation at 3,500 *g* for 10 minutes. The nuclear pellet was resuspended in low-salt buffer (20 mM HEPES [pH 7.9], 1.5 mM MgCl_2_, 20 mM KCl, 0.2 mM EDTA, 25% glycerol, 1 mM β-mercaptoethanol, 0.5 mM DTT, 0.5 mM PMSF). After slowly adding high-salt buffer (20 mM HEPES [pH 7.9], 1.5 mM MgCl_2_, 1.2 M KCl, 0.2 mM EDTA, 25% glycerol, 1 mM β-mercaptoethanol, 0.5 mM DTT, 0.5 mM PMSF), the nuclear extract was rotated for 15 minutes at 4°C. Finally, the nuclear fraction was resuspended in BC-0 buffer (20 mM Tris, pH 7.3, 0.2 mM EDTA, 20% glycerol, 1 mM β-mercaptoethanol, 0.5 mM DTT, 0.5 mM PMSF) after 10 minutes centrifugation at 13,500 *g* to remove insoluble material.

***In situ* proximity ligation assay**

*In situ* proximity ligation assay (PLA) was performed using the Duolink In Situ kit (Sigma-Aldrich, DUO92008) following the manufacturer’s product instructions. HCT116 and RKO cell lines were cultured on coverslips and fixed with 4% PFA. Cells were permeabilized with 0.5% Triton-X 100 and then blocked in Blocking Solution for 60 minutes at 37°C. The coverslips were incubated with primary antibodies against RNF138 (1:50 dilution, made in our lab) and NIBP (1:50 dilution, Proteintech, 16014-1-AP) at 4°C overnight. Rat (Santa Cruz Biotech, sc-2026) and rabbit IgG (Millipore, 12-370) were used as negative controls. Slides were then incubated for 1 hour at 37°C with premixed Duolink In Situ PLA Probe Anti-Rat MINUS (Sigma-Aldrich, DUO92010) and Duolink In Situ PLA Probe Anti-Rabbit PLUS (1:5 dilution with Duolink Antibody Diluent) (Sigma-Aldrich, DUO92008), followed by emersion in 1 × wash buffer for 5 minutes twice. The slides were then incubated in 1 × ligation buffer at 37°C for 30 minutes. After washing, the slides were incubated with 1 × amplification buffer at 37°C for 100 minutes and, finally, sealed using Duolink In Situ Mounting Medium with DAPI, and the images were captured on a fluorescence confocal microscope. The PLA signals were recognized as red fluorescent spots. PLA score was determined by normalizing the number of PLA spots counted in each sample to the average number of PLA spots counted in the control sample (IgG- IgG).

**Small-molecule inhibitor cytotoxicity assay**

To determine the effect of RNF138 on NF-κB inhibitor sensitivity, HCT116 and RKO cell lines were seeded into 96-well plates at an initial density of 2 × 10^3^ and 6 × 10^3^ cells/well, respectively. After adhering, the HCT116 and RKO cells were treated with increasing concentrations of NFκB inhibitors (IKK16, BAY11-7082, Bortezomib, and SC75741) (Selleckchem, S2882, S2913, S1013, S7273) for 72 hours. Then the cytotoxicity assay was performed with the Cell Counting Kit-8 (CCK8) (Dojindo Molecular Technology, CK04, Fukuoka, Japan) following the manufacturer’s instructions. In brief, 10 μl of CCK8 solution was added to 90 μl of complete IMDM medium and incubated at 37°C for 90 minutes. The 450 nm absorbance was measured using an ELISA plate reader, and the IC50 was determined through non-linear regression analysis by Graphpad 8.0 software.

**Cell derived xenograft models**

To investigate the antitumor effects of RNF138 *in vivo*, BALB/c nude mice were subcutaneously injected via the dorsal flank skins with 1 × 10^6^ HCT116 cells (left: RNF138^WT^, right: RNF138^KO^) in 100 μl matrigel (Corning, 354234). Fourteen animals were randomly assigned to two groups (seven mice per group) and treated with vehicle control (corn oil, Solarbio, C7030, Beijing, China) or SC75741 at a dose of 15 mg/kg via intraperitoneal injection every other day. Tumors were measured by calipers every other day, and tumor volumes were calculated using the following formula: V = (L × W^2^)/2, where V = volume (in mm^3^), L = length (in mm), and W = width (in mm). At 18-days post-implantation, the mice were euthanized and all tumors were removed, photographed, and weighed. Furthermore, parts of the tumor tissues were embedded in paraffin, sectioned, and subjected to H&E staining and IHC analysis with antibodies against RNF138, pp65, ICAM1, PTGS2, Ki67, or PCNA. The remaining tissues were snap-frozen and stored at −80°C.

**Patient-derived xenograft models**

Tumor tissues for the PDXs models were obtained from CRC patients undergoing surgery at the Cancer Hospital, Chinese Academy of Medical Sciences. Detailed information is provided in Supplementary Table 6. CRC tissues were collected in culture medium, supplemented with 1% penicillin-streptomycin, and kept on water ice for engraftment within 24 hours. Approximately 20-30 mg tissue fragments were subcutaneously implanted into NOD/SCID mice dorsal flanks. The successfully engrafted tumor models were then passaged and banked after two to three passages.

For the therapeutic studies using PDX models, the engrafted tumor models were propagated and expanded to 18 NOD/SCID mice. When the tumors were approximately ~100 mm^3^, mice were randomly assigned to a vehicle control (corn oil) or SC75741 (15 mg/kg, Selleckchem, S7273) treatment group (9 mice per group). The animals were treated by intraperitoneal injection every other day for 18 days. The tumor growth volume (mm^3^) and mouse body weight (g) were recorded every other day. Finally, mice were sacrificed and all tumors were collected, photographed, and weighed. A small portion of xenograft tumors were embedded and sectioned for H&E, RNF138, pp65, ICAM1, PTGS2, Ki67, or PCNA staining. The remaining tissues were snap frozen in liquid nitrogen and stored at −80°C.

**References**

1 Zaki, M. H. et al. The NOD-like receptor NLRP12 attenuates colon inflammation and tumorigenesis. *Cancer cell* **20**, 649-660 (2011).

2 Karki, R. et al. NLRC3 is an inhibitory sensor of PI3K-mTOR pathways in cancer. *Nature* **540**, 583-587 (2016).

3 Xue, X. & Shah, Y. M. In vitro organoid culture of primary mouse colon tumors. *J. Vis.Exp.* e50210 (2013).

4 Kesselring, R. et al. IRAK-M Expression in Tumor Cells Supports Colorectal Cancer Progression through Reduction of Antimicrobial Defense and Stabilization of STAT3. *Cancer cell* **29**, 684-696 (2016).

**Supplementary Figures and Figure Legends**

**
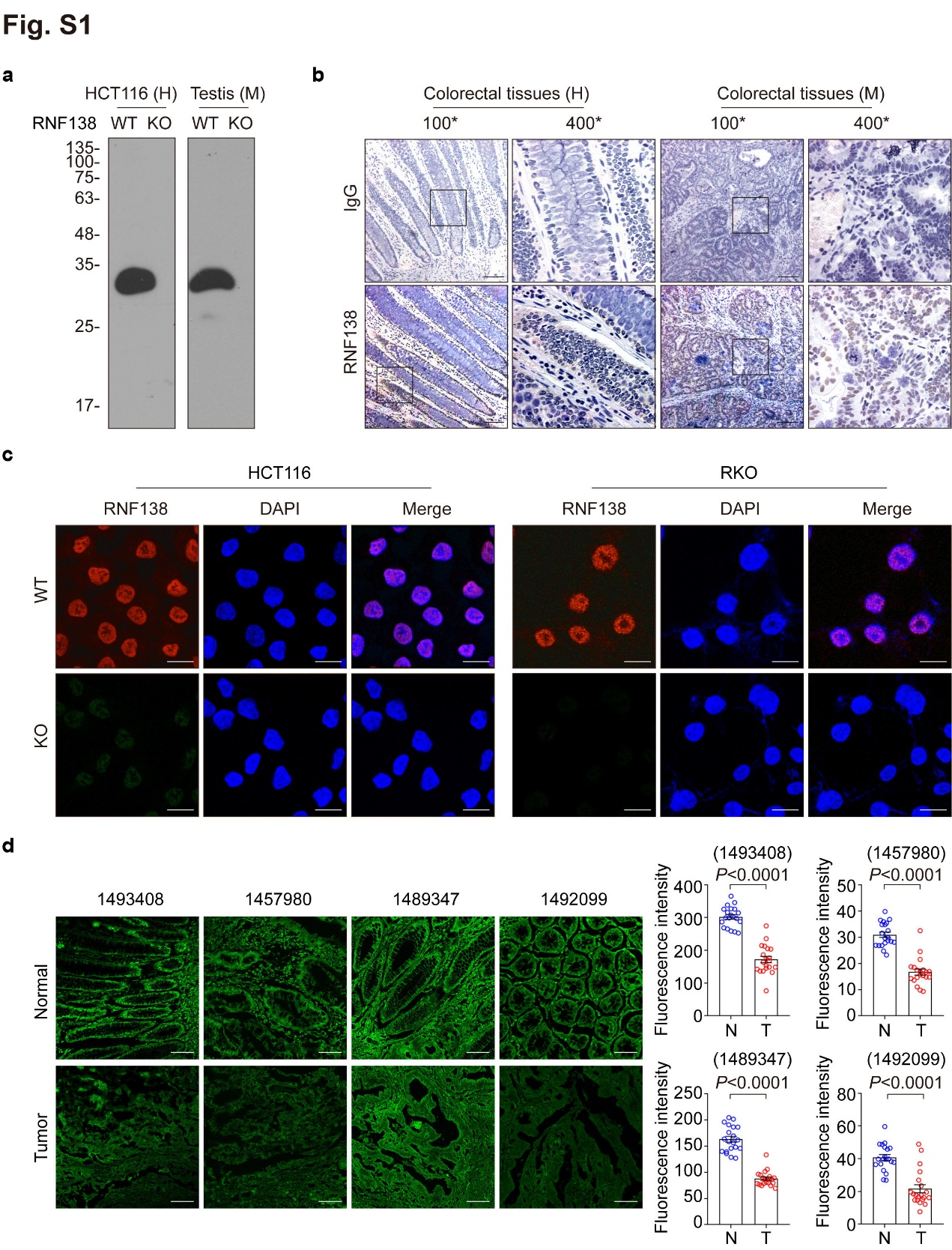
**

**Figure S1. The specificity of in-house anti-RNF138 rat monoclonal antibody is determined by immunoblotting, immunostaining and immunohistochemistry.**

(**a**) Immunoblotting analysis of the specificity of the RNF138 monoclonal antibody in wild-type and knockout human HCT116 cells and mouse testes. Antibody dilution: 1:1000. Sample loading: 20 μg. (**b**) [Immunohistochemical](javascript:;) staining with IgG or RNF138 monoclonal antibody of human (left) and mouse (right) colorectal tissue sections. Antibody dilution: 1:100. Scale bar, 100 μm. (**c**) Immunofluorescence staining for RNF138 (red), with DAPI staining of nuclei (blue), in HCT116 and RKO RNF138^WT^ (top) and RNF138^KO^ (bottom) cells. Antibody dilution: 1:100. Scale bar, 20 μm. (**d**) Representative immunofluorescence staining for RNF138 (green) in human CRC and normal tissues from four patients (left). Quantification of anti-RNF138 staining density in CRC (T) or adjacent normal tissues (N) (right). Data are mean ± SEM. Scale bar, 200 μm.

**
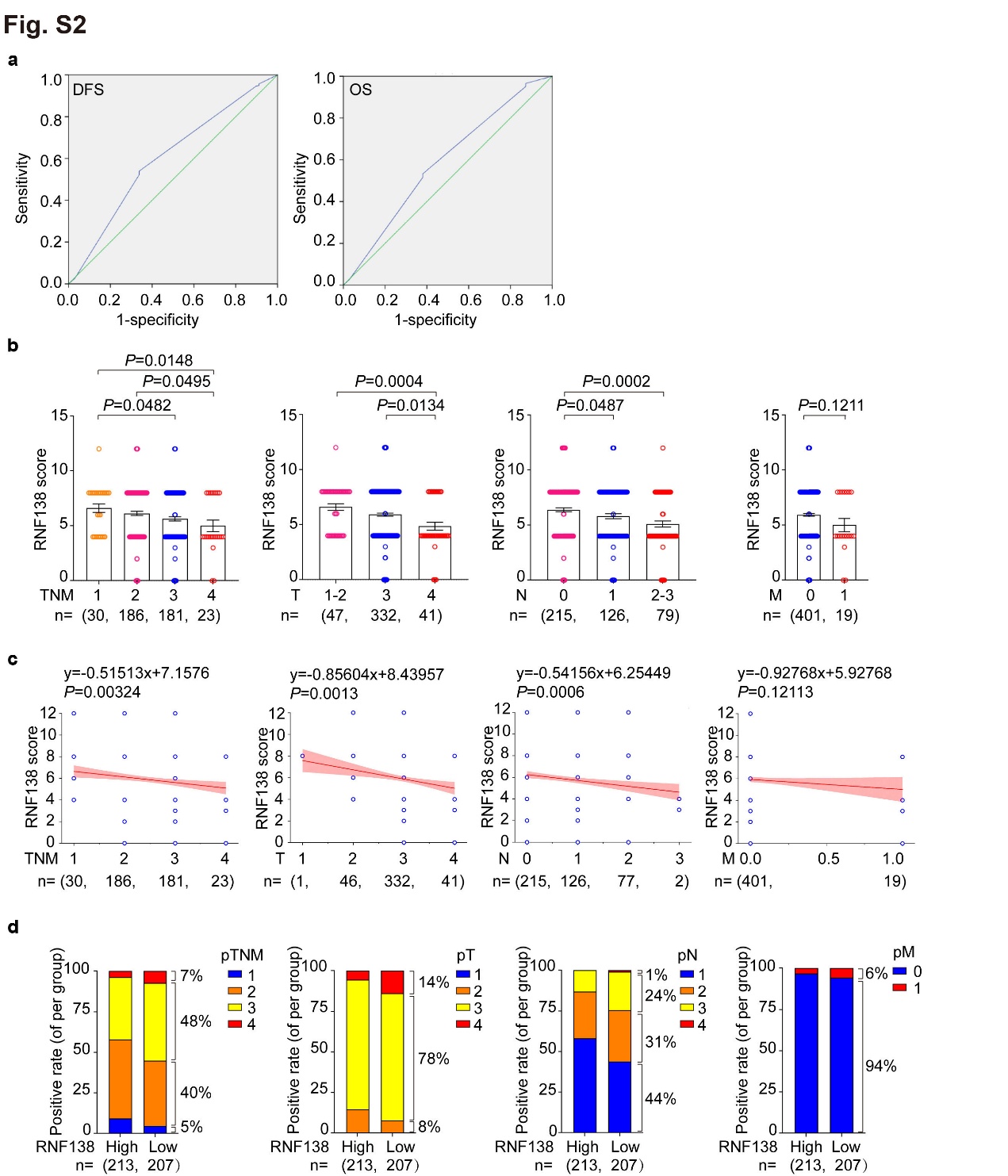
**

**Figure S2. The correlation between RNF138 and human CRC clinical parameters is evaluated in 420 TMAs.**

(**a**) RNF138 cut-off value was determined by ROC curves of DFS and OS survival in 420 CRC patients. (**b**) Quantification of RNF138 expression in pTNM (1, 2, 3, 4), pT (1-2, 3, 4), pN (0, 1, 2-3), and pM (0, 1) stages in 420 CRC patients. Data are mean ± SEM. (**c**) Correlation plot of RNF138 IHC staining and pTNM (1, 2, 3, 4), pT (1, 2, 3, 4), pN (0, 1, 2, 3), and pM (0, 1) stages in 420 CRC patients. Correlation was evaluated using nonparametric Spearman’s test. The formula and *p*-value are indicated. (**d**) Percentages of pTNM (1, 2, 3, 4), pT (1, 2, 3, 4), pN (0, 1, 2, 3), and pM (0, 1) stages in RNF138^high^ (n = 213) and RNF138^low^ (n = 207) groups of 420 CRC patients.


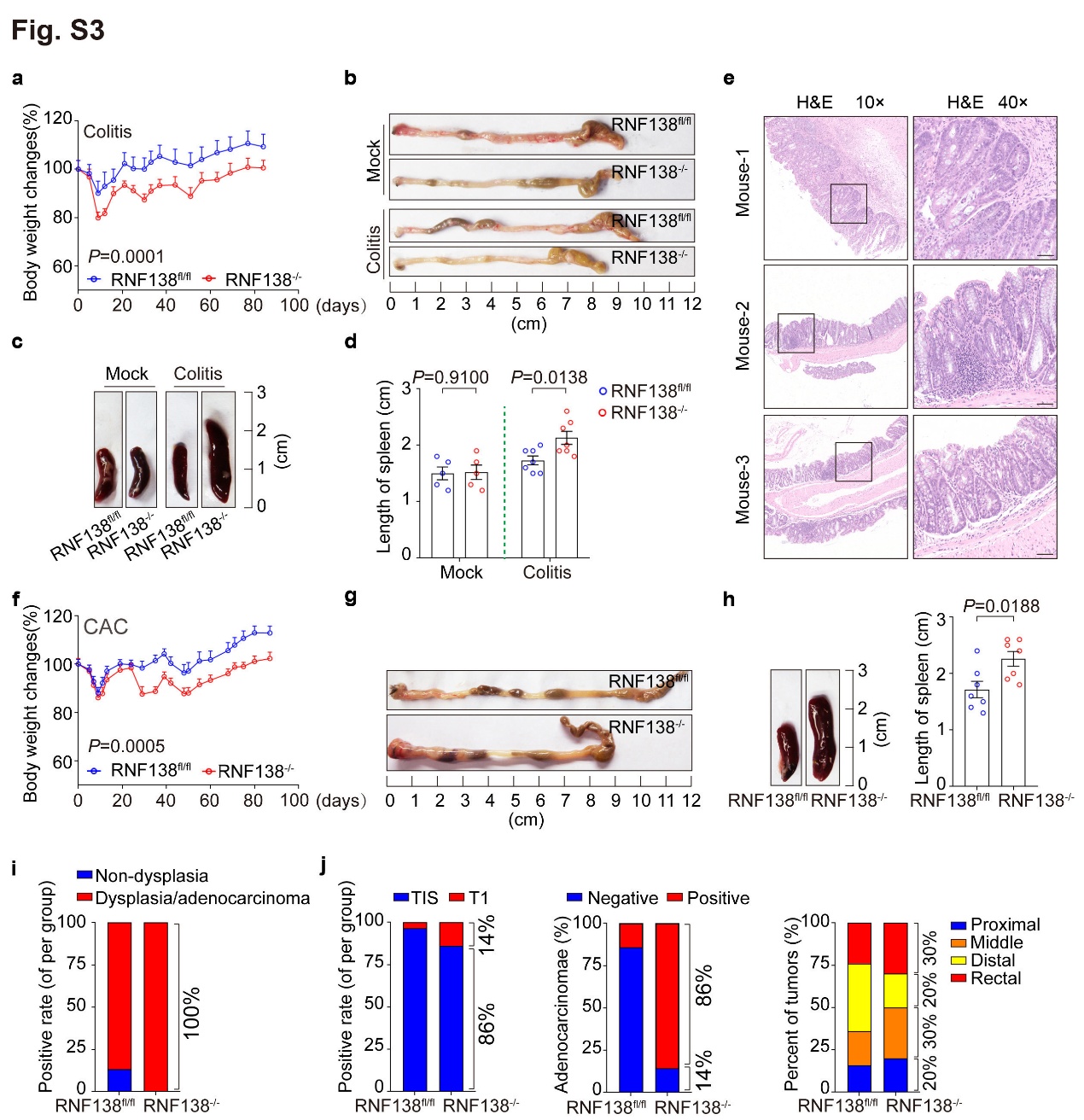


**Figure S3. RNF138^-/-^ mice display increased chronic colitis and tumorigenesis upon DSS and AOM/DSS administration.**

#### (a) Body weight changes during chronic colitis with DSS treatment (RNF138^fl/fl^, n = 15; RNF138^-/-^, n = 23). (b) Representative gross morphology of colon in mock and chronic colitis model mice. (c-d) Representative gross morphology (c) and quantification of spleen length (d) in mock (n = 5) and chronic colitis (n = 7) model mice. (e) H&E staining of three other precancerous lesions after DSS administration in RNF138^-/-^ mice. Scale bar, 100 μm. (f) Body weight changes during the course of the CAC model (RNF138^fl/fl^, n = 17; RNF138^-/-^, n = 23). (g) Representative gross images of colons at autopsy after AOM/DSS administration. (h) Representative gross images (left) and quantification (right) of spleens from CAC model (n = 7). (i) Quantitation of tumor formation frequency in RNF138^fl/fl^ and RNF138^-/-^ CAC models. (j) Percentages of invasive stage (TIS and T1), adenocarcinoma formation rate, and tumor location (proximal, middle, distal colon, rectum) in RNF138^fl/fl^ and RNF138^-/-^ CAC model mice. Data are represented as mean ± SEM.

**
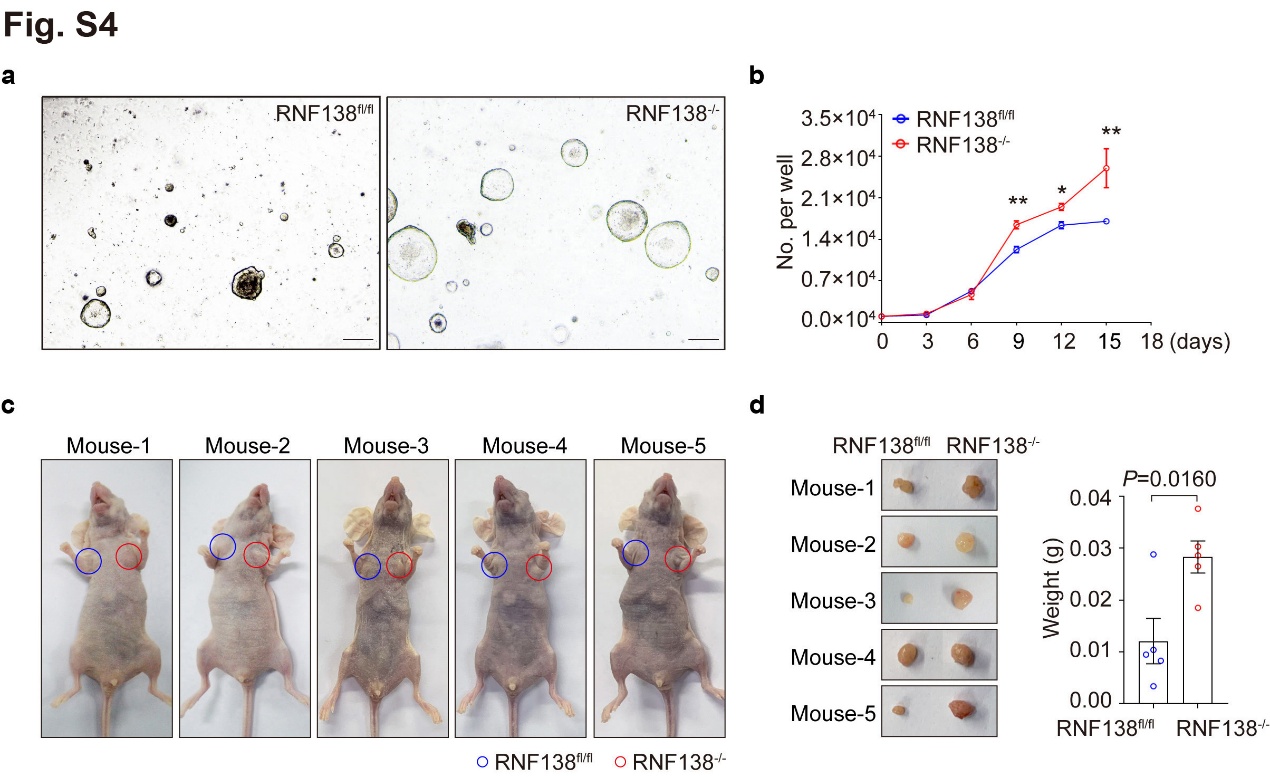
**

#### Figure S4. RNF138^-/-^ mice display increased tumor proliferation in mouse organoids derived from CAC model.

#### (a) Representative images of cultured organoids in matrigel from RNF138^fl/fl^ and RNF138^-/-^ mice derived from AOM/DSS model on 9th day. Scale bar, 100 μm. (b) Proliferation of murine tumor organoids of RNF138^fl/fl^ and RNF138^-/-^ (n = 5). (c) Gross images of tumor organoid grafts from RNF138^fl/fl^ and RNF138^-/-^ mice on 60th day after implantation. (d) Tumor organoid grafts and quantification of tumor weights from RNF138^fl/fl^ and RNF138^-/-^ mice at experimental endpoint (n = 5). Data are mean ± SEM (n = 5). Data are represented as mean ± SEM. *, *p* < 0.05 and **, *p* < 0.01.

**
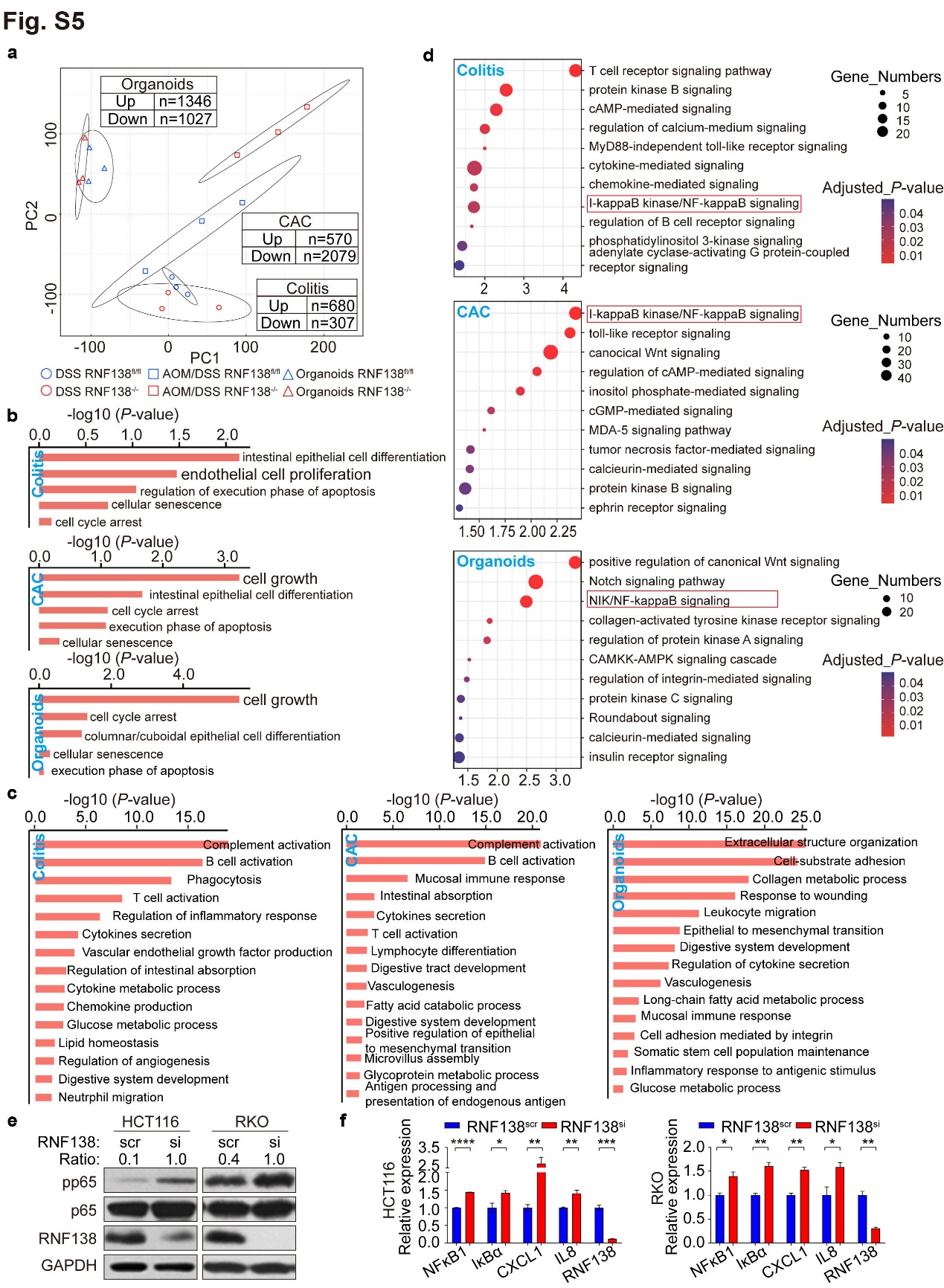
**

**Figure S5. NF-κB signaling pathway is overactivated in RNF138^-/-^ colitis, CAC and CAC-derived organoids groups by transcriptomic analysis.**

(**a**) Principal component analysis (PCA) plots of mRNA expression levels in RNF138^fl/fl^ and RNF138^-/-^ groups of colitis, CAC, and organoid mice models. The significantly differentiated gene numbers (padj < 0.05, fold change ≥ 2) are marked on the plot. (**b**) Enrichment of gene ontology (GO) terms of cell growth, apoptosis, cell cycle, differentiation, and senescence. *P*-values are presented in the bar graph. (**c**) Enrichment of GO terms (biological processes) in RNF138^fl/fl^ compared with RNF138^-/-^ clusters from DAVID informatics. (**d**) The top 10 significant signaling pathways in RNF138^fl/fl^ and RNF138^-/-^ groups of colitis, CAC, and organoid models are shown *via* bubble plots. NF-κB signaling is marked by red box. (**e**) Immunoblotting analysis of phosphorylation and total p65 in HCT116 and RKO cells transfected with RNF138 or control siRNA. The densitometric values were normalized to *RNF138*-silenced cells. (**f**) qPCR analysis of NF-κB target genes (*NFκB1*, *IκBα*, *CXCL1*, and *IL8*) mRNA expression levels in HCT116 and RKO cells transfected with RNF138 or control siRNA. ACTB was used as an internal control. Data were normalized to CRC cells transfected with control siRNA. Data are mean ± SEM (n = 3). *, *p* < 0.05; **, *p* < 0.01; ***, *p* < 0.001 and ****, *p* < 0.0001.

**
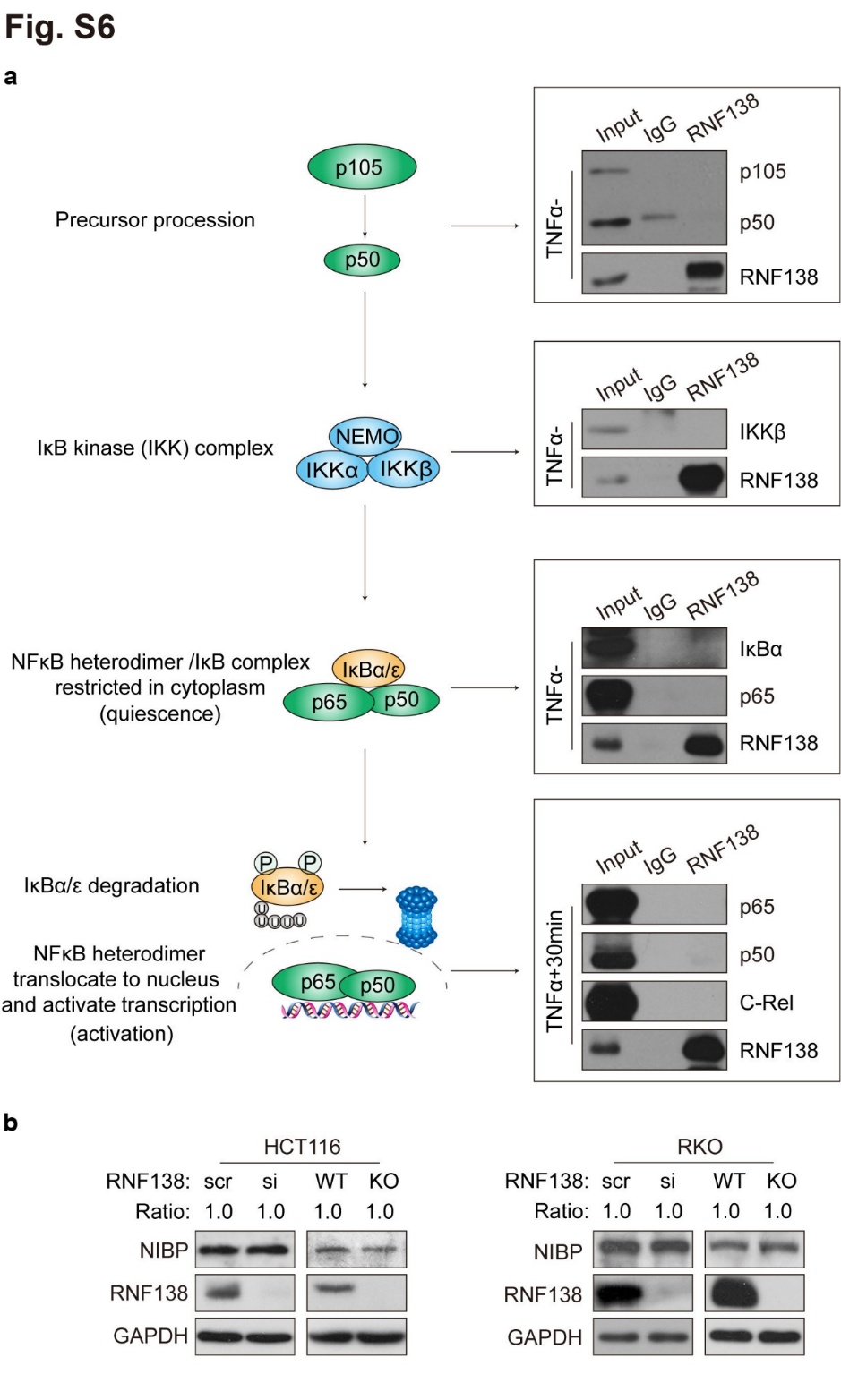
**

**Figure S6. The interaction between RNF138 and NF-κB signaling components is analyzed by co-immunoprecipitation.**

(**a**) Schematic overview of four critical stages of classical NF-κB pathway activation and core proteins (left). Co-immunoprecipitation analysis of endogenous RNF138 and core protein interaction in HCT116 cell lysates (right). (**b**) Immunoblotting analysis of NIBP in HCT116 and RKO cells transfected with RNF138 or control siRNA in RNF138 wild-type or knockout cells. GAPDH served as a loading control.

**
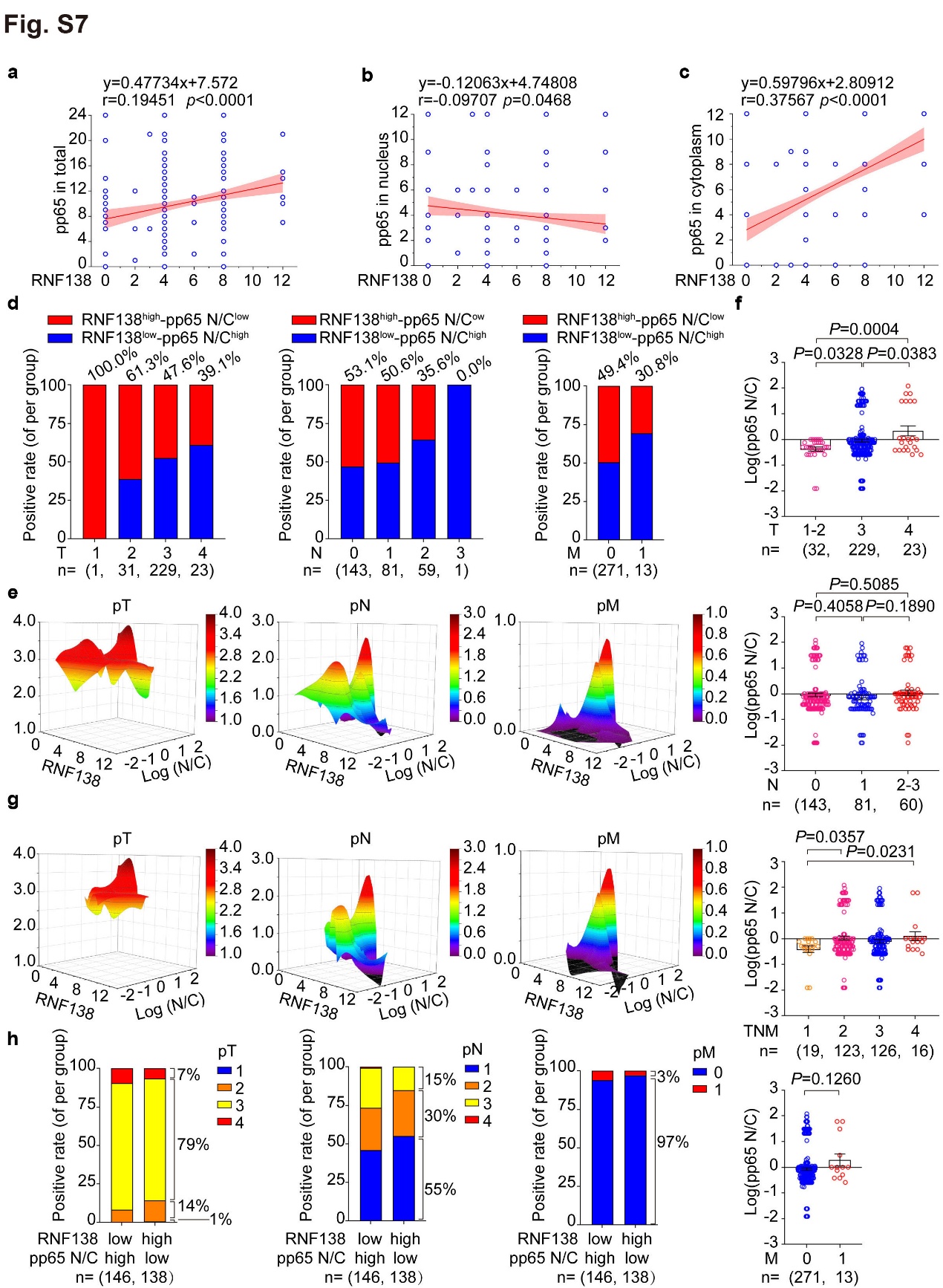
**

**Figure S7. RNF138 ablation accords with aberrant activation of NF-кB signaling in tumors correlates with unfavorable clinic outcomes.**

(**a**-**c**) Correlation between RNF138 IHC staining and total (a), nuclear (b), and cytoplasmic (c) pp65 IHC scores by Pearson’s test (n = 420). The formula, coefficient of correlation (r), and *p*-value are indicated (n = 420). (**d**) Percentages of RNF138^high^-pp65 N/C ratio^low^ (red) and RNF138^low^-pp65 N/C ratio^high^ (blue) in pT (1, 2, 3, 4), pN (0, 1, 2, 3), and pM (0, 1) stages (n = 284). (**e**) Correlation among RNF138, log(pp65N/C), and pT (1, 2, 3, 4), pN (0, 1, 2, 3), and pM (0, 1) stages in 420 CRC patients. Log(N/C), log(pp65N/C). (**f**) Quantification of log(pp65N/C) expression in pTNM (1, 2, 3, 4), pT (1-2, 3, 4), pN (0, 1, 2-3), and pM (0, 1) stages in 284 CRC patients. Data are mean ± SEM. (**g**) Correlation among RNF138, log(pp65N/C) and pT (1, 2, 3, 4), pN (0, 1, 2, 3), and pM (0, 1) stages in 284 CRC patients. Log(N/C), log(pp65N/C). (**h**) Percentages of pT (1, 2, 3, 4), pN (0, 1, 2, 3), and pM (0, 1) stages in RNF138^high^-pp65 N/C ratio^low^ (n = 138) and RNF138^low^-pp65 N/C ratio^high^ (n = 146) CRC patient TMAs.

**
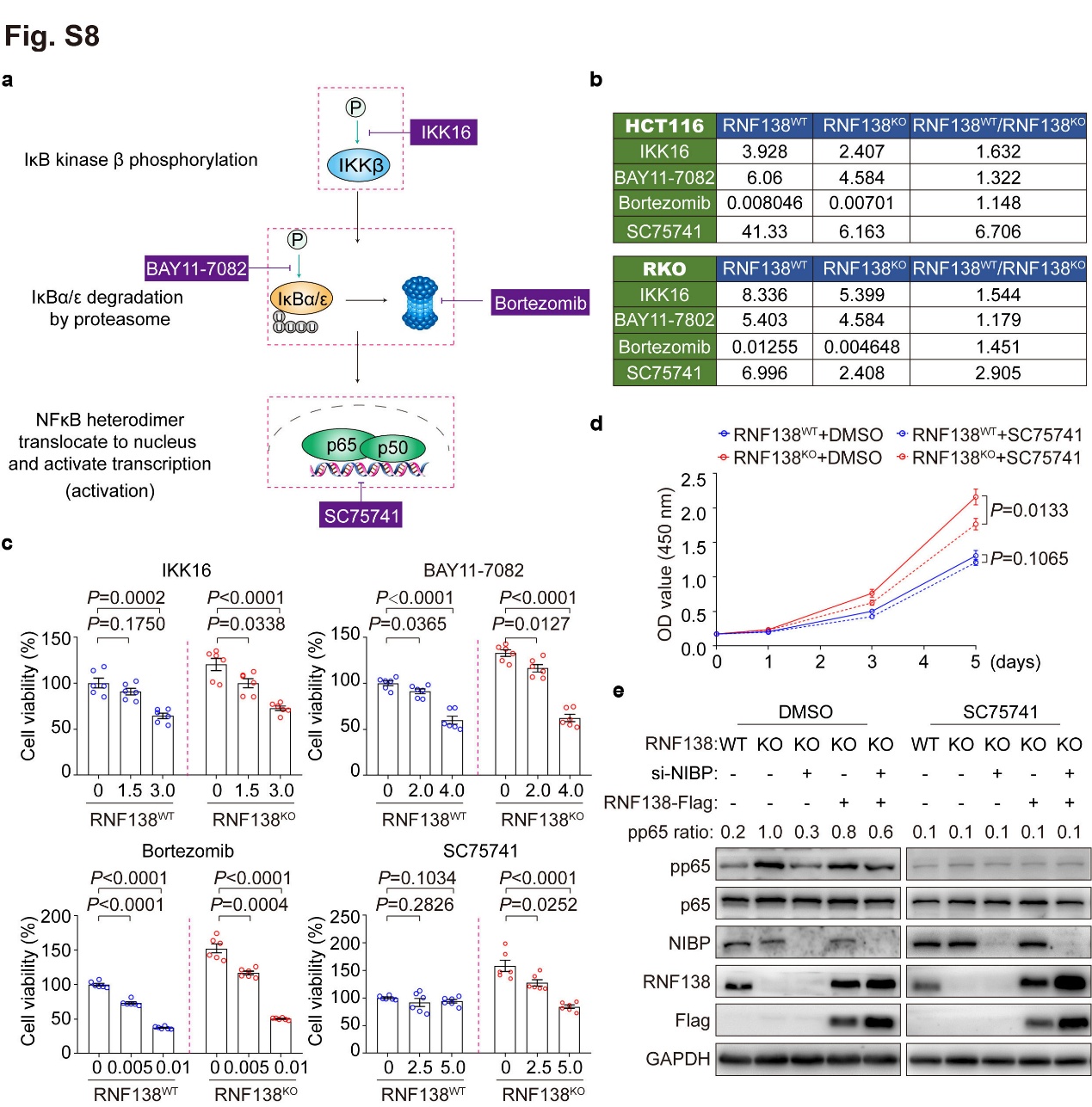
**

**Figure S8. The inhibitory impact of SC75741 on NF-κB signaling is addressed in RNF138-knockout CRC cells.**

(**a**) Schematic mechanisms of IKK16, BAY11-7082, Bortezomib, and SC75741 in classical NF-κB pathway. (**b**) IC50 values of four inhibitors (IKK16, BAY11-7082, Bortezomib, and SC75741) in HCT116 and RKO of RNF138^WT^ and RNF138^KO^ cells. Ratio, IC50^WT^/IC50^KO^. (**c**) Cell viability analysis of HCT116 cells of RNF138^WT^ and RNF138^KO^ treated with vehicle or indicated dose of IKK16, BAY11-7082, Bortezomib, and SC75741 inhibitors. Data are mean ± SEM (n = 7). (**d**) Cell growth of RNF138^WT^ and RNF138^KO^ HCT16 cells treated with DMSO or SC75741. Data are mean ± SEM (n = 7). (**e**) Immunoblotting analysis of phosphorylation and total p65 and IKKβ in RNF138^WT^ and RNF138^KO^ HCT116 cells transfected with NIBP or control siRNA for 24 h, then transfected with EV or RNF138-Flag plasmid for 24 h and treated with vehicle or SC75741 for a further 24 h. GAPDH was used as an internal loading control.

**
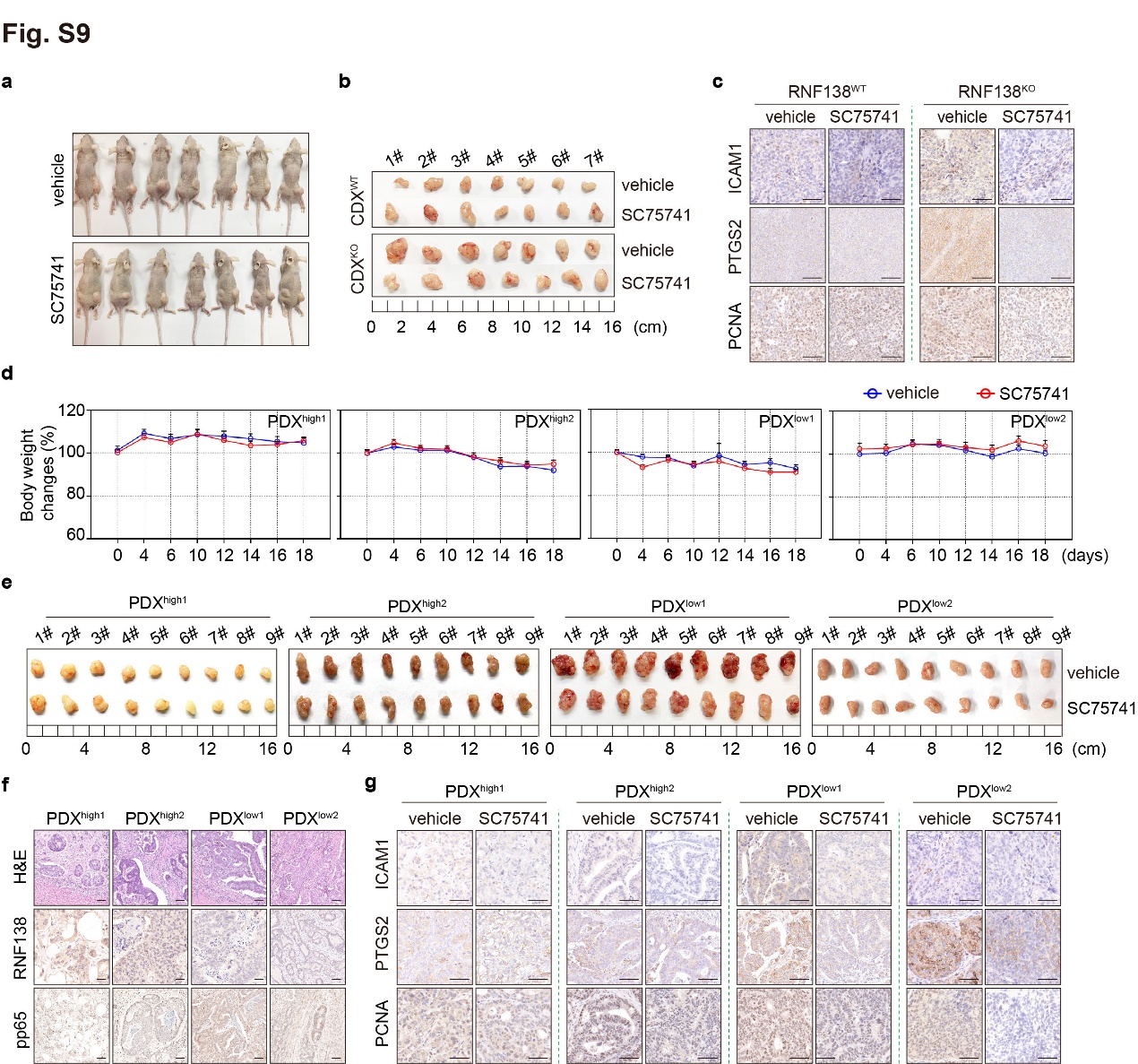
**

**Figure S9. The inhibitory effect of SC75741 on NF-κB signaling is determined in RNF138 suppressed CDX and PDX models.**

(**a**) Gross xenograft images from RNF138^WT^ (left) and RNF138^KO^ (right) HCT116 cells treated with vehicle or SC75741 by intraperitoneal injection. (**b**) Tumor mass from CDX models 18 days post-implantation (n = 7). (**c**) Representative ICAM1, PTGS2, and PCNA [immunohistochemical](javascript:;) staining of tumors isolated from CDX therapy models. Scale bar, 25 μm. (**d**) Body weight percentage changes of PDX mice given vehicle or SC75741 treatment. Data are mean ± SEM (n = 9). (**e**) Tumor mass from PDX models on the 18th day post-injection SC75741 (n = 9). PDX^high1^, RNF138^high^-pp65 N/C ratio^low^; PDX^high2^, RNF138^high^-pp65 N/C ratio^low^; PDX^low1^, RNF138^low^-pp65 N/C ratio^high^, PDX^low2^, RNF138^low^-pp65 N/C ratio^high^. (**f**) Representative H&E and [immunohistochemical](javascript:;) staining of RNF138 and pp65 in patient tissue sections. Scale bar, 25 μm. (**g**) Representative ICAM1, PTGS2, and PCNA [immunohistochemical](javascript:;) staining of tumors collected from four PDX mice therapy models. Scale bar, 25 μm.

**
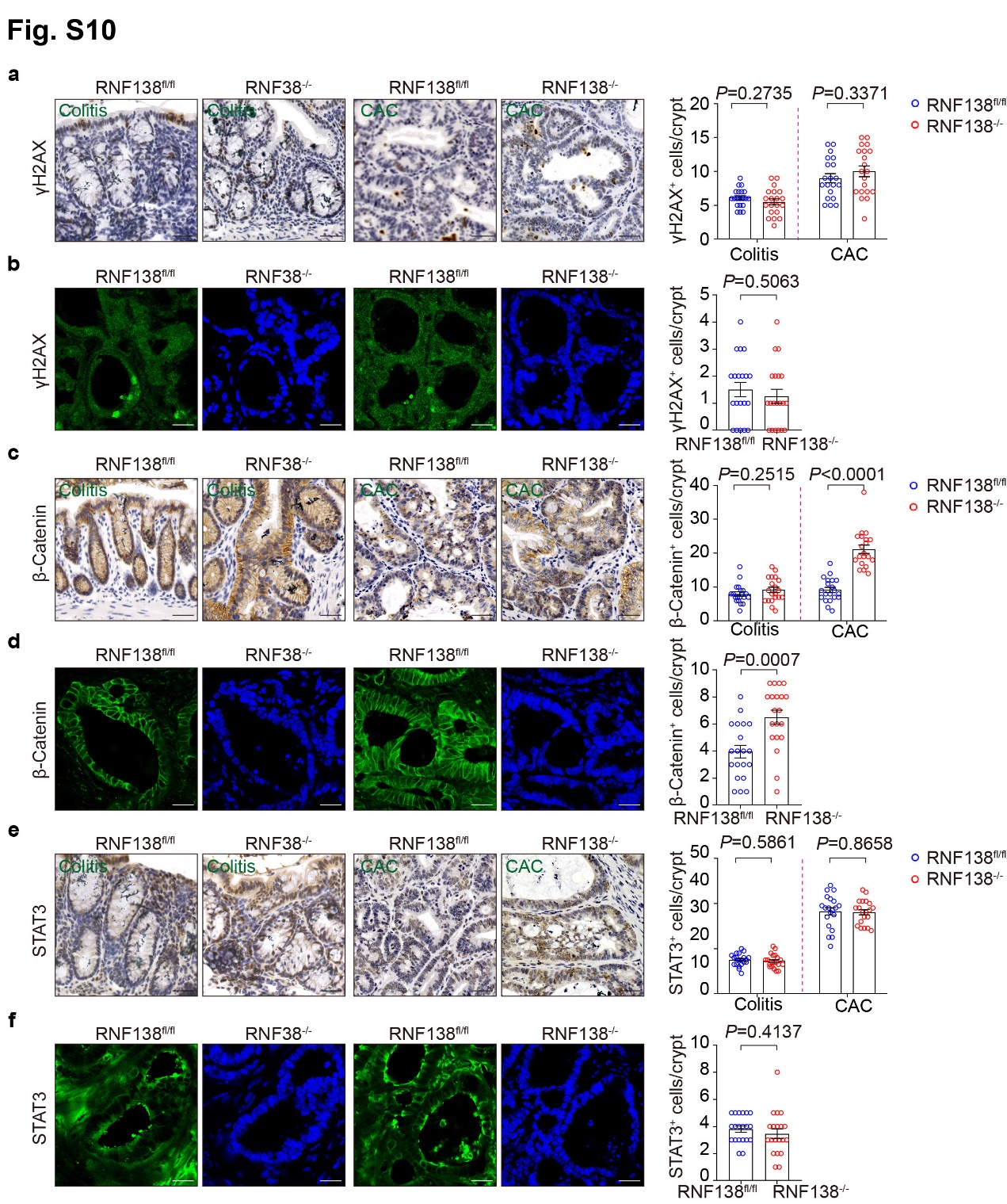
**

**Figure S10. The activation of Wnt, JAK-STAT3, and DNA damage signaling is assessed between RNF138^fl/fl^ and RNF138^-/-^ groups in colitis, CAC, and organoid models.**

(**a**) Representative [IHC](javascript:;) staining (left) and quantification (right) of γH2AX positive cells per crypt in colitis and CAC models. Scale bar, 25 μm. (**b**) Representative immunofluorescence staining (left) and quantification (right) of γH2AX (green)-positive cells per crypt in organoid models. Scale bar, 20 μm. (**c**, **e**) Representative IHC staining (left) and quantification (right) of nuclear β-Catenin (c) and nuclear STAT3 (e) in colitis and CAC models (n = 20). Scale bar, 25 μm. (**d**, **f**) Representative immunofluorescence staining (left) and quantification (right) of nuclear β-Catenin (d) and nuclear STAT3 (f) in organoid models (n = 20). Scale bar, 20 μm. Data are represented as mean ± SEM.


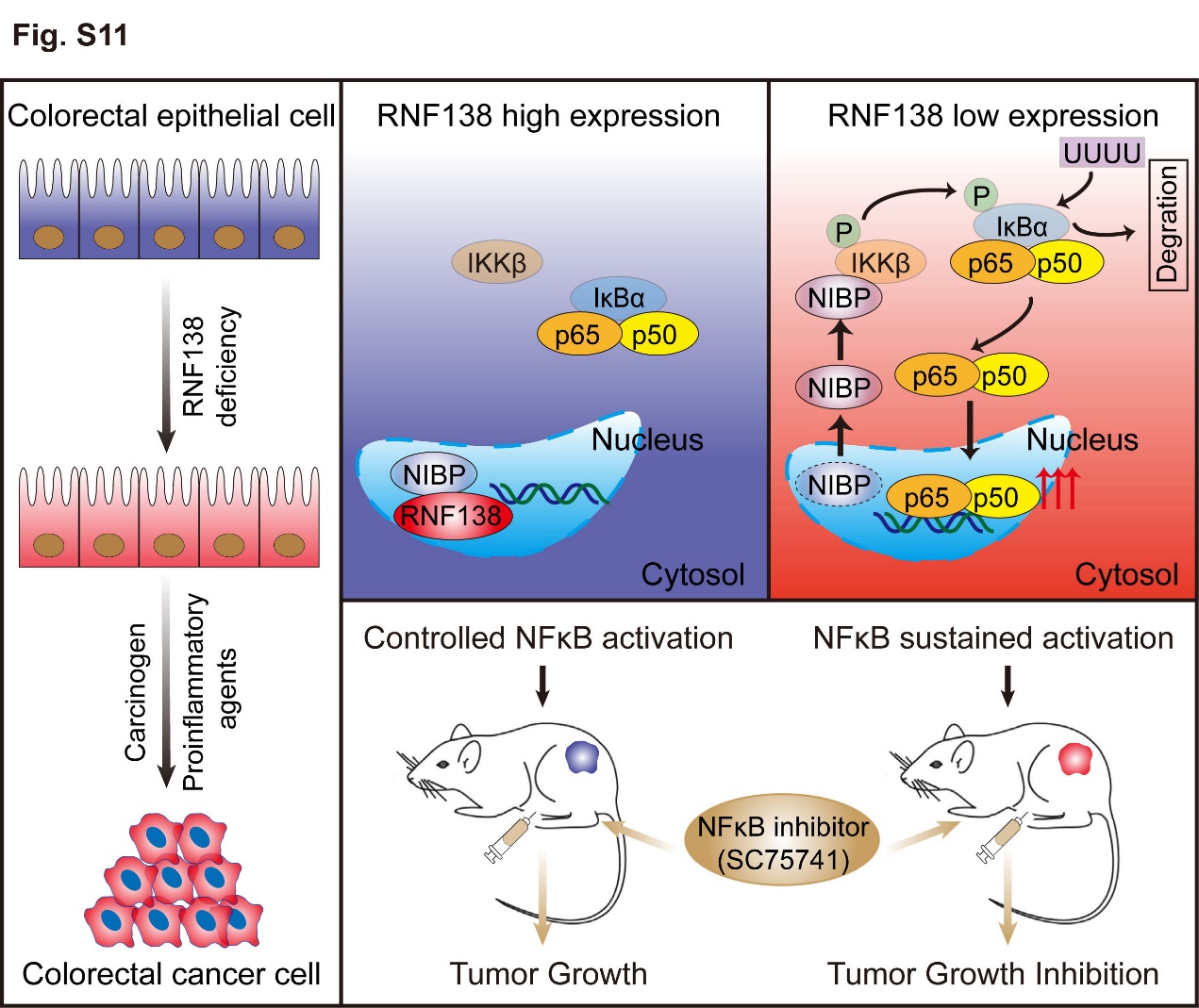


**Figure S11. Schematic overview for the molecular basis of RNF138 as a promising target for CRC treatment.**

**Supplementary Tables**

**Table S1. Summary of TCGA RNA-seq data used in this study.**

| Cancer type | Abbreviation | Normal | Numbers | Tumor | Numbers | *P* value |
| --- | --- | --- | --- | --- | --- | --- |
| colorectal adenocarcinoma | CRC | 6.90 | 51 | 6.35 | 377 | <0.0001* |
| Skin cutaneous Melanoma | SKCM | 6.570 | 1 | 5.81 | 102 | - |
| Liver hepatocellular carcinoma | LIHC | 6.27 | 50 | 5.85 | 371 | <0.0001* |
| Cholangiocarcinoma | CHOL | 6.29 | 8 | 5.88 | 36 | 0.0011* |
| Thyroid carcinoma | THCA | 6.22 | 59 | 5.95 | 504 | <0.0001* |
| Pancreatic adenocarcinoma | PAAD | 6.29 | 4 | 6.00 | 178 | 0.1395 |
| Head and neck squamous cell carcinoma | HNSC | 6.19 | 44 | 6.03 | 520 | 0.0250* |
| Thymoma | THYM | 6.78 | 2 | 6.54 | 120 | 0.3821 |
| Uterine corpus endometrial carcinoma | UCEC | 6.44 | 24 | 6.33 | 176 | 0.2690 |
| Lung adenocarcinoma | LUAD | 6.38 | 59 | 6.30 | 515 | 0.1291 |

*The signiﬁcance of RNF138 FPKM expression were calculated by unpaired two-tailed Student's t-test for 10 kinds of adjacent normal and tumor tissues.

a. CRC, colorectal adenocarcinoma (combining COAD and READ projects); SKCM, skin cutaneous melanoma; LIHC, liver hepatocellular carcinoma; CHOL, cholangiocarcinoma, THCA, thyroid carcinoma; PAAD, pancreatic adenocarcinoma; HNSC, head and neck squamous cell carcinoma; THYM, thymoma; UCEC, uterine corpus endometrial carcinoma; LUAD, lung adenocarcinoma.

**Table S2. RNF138 mRNA expression is determined in 20 pairs’ CRC samples by qPCR.**

| No | | Age | Gender | | Normal (Mean±SD) | | Tumor (Mean±SD) | | *P* value | |
| --- | --- | --- | --- | --- | --- | --- | --- | --- | --- | --- |
| 1 | | 54 | Female | | 1.00 ± 0.02765 | | 0.41 ± 0.01384 | | 0.0013* | |
| 2 | | 65 | Male | | 1.00 ± 0.07859 | | 0.15 ± 0.00563 | | 0.0049* | |
| 3 | | 67 | Female | | 1.00 ± 0.01830 | | 0.63 ± 0.00547 | | 0.0013* | |
| 4 | | 75 | Female | | 1.00 ± 0.05128 | | 0.29 ± 0.01276 | | 0.0020* | |
| 5 | | 40 | Male | | 1.00 ± 0.02487 | | 0.15 ± 0.00049 | | 0.0004* | |
| 6 | | 78 | Male | | 1.00 ± 0.00866 | | 0.29 ± 0.06494 | | 0.0044* | |
| 7 | | 72 | Female | | 1.00 ± 0.01132 | | 0.68 ± 0.02117 | | 0.0035* | |
| 8 | | 64 | Male | | 1.00 ± 0.04881 | | 0.31 ± 0.01910 | | 0.0041* | |
| 9 | | 46 | Male | | 1.00 ± 0.01132 | | 0.35 ± 0.01928 | | 0.0002* | |
| 10 | | 86 | Male | | 1.00 ± 0.13575 | | 0.73 ± 0.01152 | | 0.0474* | |
| 11 | | 70 | Male | | 1.00 ± 0.13052 | | 0.46 ± 0.10596 | | 0.0105* | |
| 12 | | 71 | Female | | 1.00 ± 0.02556 | | 0.26 ± 0.12312 | | 0.0012* | |
| 13 | | 72 | Female | | 1.00 ± 0.24581 | | 0.16 ± 0.01779 | | 0.0380* | |
| 14 | | 65 | Male | | 1.00 ± 0.31403 | | 0.79 ± 0.08928 | | 0.2873 | |
| 15 | | 78 | Female | | 1.00 ± 0.10235 | | 0.04 ± 0.00143 | | 0.0057* | |
| 16 | | 67 | Male | | 1.00 ± 0.13269 | | 0.38 ± 0.01539 | | 0.0179* | |
| 17 | | 65 | Male | | 1.00 ± 0.05729 | | 0.24 ± 0.03634 | | 0.0066* | |
| 18 | | 53 | Female | | 1.00 ± 0.00104 | | 0.66 ± 0.00835 | | 0.0178* | |
| 19 | | 84 | Male | | 1.00 ± 0.14491 | | 0.38 ± 0.02084 | | 0.1255 | |
| 20 | | 56 | Female | | 1.00 ± 0.07534 | | 0.21 ± 0.22942 | | 0.0418* | |
|  |  |  |  | |  | |  | |  |  |

*The signiﬁcance of RNF138 mRNA expression was calculated by two-tailed paired Student's t-test for 20 pairs of adjacent normal tissues and tumor tissues.

**Table S3. Characteristics of the 134 CRC patients.**

| Source | | N | Normal | Tumor | *P* value |
| --- | --- | --- | --- | --- | --- |
| Colon | | 67 | 9.19±3.45 | 7.31±2.88 | <0.0001* |
| Gender | Male | 41 (61.19%) | 9.49±3.34 | 7.61±2.66 |  |
|  | Female | 26 (38.81%) | 8.73±3.57 | 6.85±4.20 |  |
| Age | ≤65 | 35 (52.24%) | 8.71±3.76 | 6.91±3.17 |  |
|  | ＞65 | 30 (44.78%) | 8.73±3.57 | 6.85±4.20 |  |
|  | Unknown | 2 (2.99%) | 10.0±2.0 | 8.00±0.0 |  |
| Size (cm) | ＜5 | 30 (44.78%) | 9.57±3.28 | 7.23±2.73 |  |
|  | ≥5 | 37 (55.22%) | 8.89±3.55 | 7.38±2.99 |  |
| Tumor Location | Right and transverse colon | 26 (38.81%) | 8.81±3.84 | 7.35±3.38 |  |
|  | Left colon | 41 (61.19%) | 9.44±3.15 | 7.29±2.50 |  |
| Pathological type | [mucinous adenocarcinoma](http://dict.cnki.net/dict_result.aspx?searchword=%e7%b2%98%e6%b6%b2%e8%85%ba%e7%99%8c&tjType=sentence&style=&t=mucinous+adenocarcinoma) | 8 (11.94%) | 9.13±3.44 | 7.5±3.12 |  |
|  | [Tubular adenocarcinoma](javascript:;) | 20 (29.85%) | 10.6±2.35 | 8.4±2.15 |  |
|  | [signet ring cell carcinoma](javascript:showjdsw('showjd_0','j_0')) | 3 (4.48%) | 6.0±4.32 | 4.67±5.19 |  |
|  | Adenocarcinoma  (unknown) | 36 (53.73%) | 8.69±3.57 | 6.89±2.64 |  |
| Rectum | | 67 | 7.21±3.64 | 6.43±2.09 | 0.0040* |
| Gender | Male | 41 (61.19%) | 7.20±3.46 | 6.34±1.97 |  |
|  | Female | 26 (38.81%) | 7.23±3.92 | 6.58±2.26 |  |
| Age | ≤65 | 39 (58.21%) | 7.33±3.71 | 6.23±2.21 |  |
|  | ＞65 | 27 (40.30%) | 6.85±3.47 | 6.67±1.89 |  |
|  | Unknown | 1 (1.49%) | 12.0 | 8.0 |  |
| Size (cm) | ＜5 | 31 (46.27%) | 6.84±3.47 | 6.19±2.23 |  |
|  | ≥5 | 35 (52.34%) | 7.57±3.74 | 6.6±1.94 |  |
|  | Unknown | 1 (1.49%) | 6.0 | 8.0 |  |
| Tumor Location | Rectum (upside) | 5 (7.46%) | 8.0±3.35 | 7.2±1.6 |  |
|  | Rectum (down) | 1 (1.49%) | 6.0 | 4.0 |  |
|  | Rectum (unknown) | 61 (91.04%) | 7.16±3.69 | 6.41±2.11 |  |
| Pathological type | [mucinous adenocarcinoma](http://dict.cnki.net/dict_result.aspx?searchword=%e7%b2%98%e6%b6%b2%e8%85%ba%e7%99%8c&tjType=sentence&style=&t=mucinous+adenocarcinoma) | 5 (7.46%) | 4.8±1.6 | 5.6±1.96 |  |
|  | [Tubular adenocarcinoma](javascript:;) | 7 (10.45%) | 9.14±3.68 | 7.43±1.40 |  |
|  | Adenocarcinoma  (unknown) | 55 (82.09%) | 7.18±3.63 | 6.38±2.13 |  |

*The signiﬁcance of RNF138 IHC staining scores was calculated by two-tailed paired Student's t-test for pairs of adjacent normal tissues and CRC tumor tissues.

**Table S4. The association between RNF138 expression and clinicopathological variables in 420 colorectal cancer patients.**

| Clinicopathological variables | N | RNF138 expression | | *P* value |
| --- | --- | --- | --- | --- |
|  |  | Negative Positive | |  |
| Gender |  |  |  | 0.307 |
| Male | 237 (56.43%) | 122 | 115 |  |
| Female | 183 (43.57%) | 85 | 98 |  |
| Age |  |  |  | 0.217 |
| ≤65 | 324 (77.14%) | 165 | 159 |  |
| ＞65 | 96 (22.86%) | 42 | 54 |  |
| Size(cm) |  |  |  | 0.388 |
| ＜5 | 222 (52.86%) | 105 | 117 |  |
| ≥5 | 198 (47.14%) | 102 | 96 |  |
| Tumor Location |  |  |  | 0.531 |
| Right and transverse colon | 103 (24.52%) | 48 | 55 |  |
| Left colon | 317 (75.48%) | 159 | 158 |  |
| Pathological type |  |  |  | 0.053 |
| mucous secretion | 33 (7.86%) | 12 | 21 |  |
| [mucinous adenocarcinoma](http://dict.cnki.net/dict_result.aspx?searchword=%e7%b2%98%e6%b6%b2%e8%85%ba%e7%99%8c&tjType=sentence&style=&t=mucinous+adenocarcinoma) | 18 (4.29%) | 6 | 12 |  |
| ulcerative adenocarcinoma | 88 (20.95%) | 43 | 45 |  |
| Raised adenocarcinoma | 127 (30.24%) | 59 | 68 |  |
| [signet ring cell carcinoma](javascript:showjdsw('showjd_0','j_0')) | 8 (1.90%) | 7 | 1 |  |
| others | 146 (34.76%) | 80 | 66 |  |
| Differentiation |  |  |  | 0.262 |
| Low | 61 (14.52%) | 29 | 32 |  |
| Moderate | 328 (78.10%) | 164 | 164 |  |
| High | 21 (5.00%) | 7 | 14 |  |
| Unkown | 10 (2.38%) | 7 | 3 |  |
| pTNM |  |  |  | 0.027* |
| 1 | 30 (7.14%) | 10 | 20 |  |
| 2 | 186 (44.29%) | 83 | 103 |  |
| 3 | 181 (43.10%) | 99 | 82 |  |
| 4 | 23 (5.48%) | 15 | 8 |  |
| pT |  |  |  | 0.003* |
| T1 | 1 (0.24%) | 0 | 1 |  |
| T2 | 46 (10.95%) | 16 | 30 |  |
| T3 | 332 (79.05%) | 162 | 170 |  |
| T4 | 41 (9.76%) | 29 | 12 |  |
| pN |  |  |  | 0.003* |
| N0 | 215 (51.19%) | 91 | 124 |  |
| N1 | 126 (30.00%) | 65 | 61 |  |
| N2 | 77 (18.33%) | 49 | 28 |  |
| N3 | 2 (0.48%) | 2 | 0 |  |
| pM |  |  |  | 0.246 |
| M0 | 401 (95.48%) | 195 | 206 |  |
| M1 | 19 (4.52%) | 12 | 7 |  |

a. pTNM, pathological tumor-node-metastasis; pT, pathological tumor; pN, pathological node; pM, pathological metastasis.

*The signiﬁcance of RNF138 expression in clinicopathological variables were calculated by χ2 test and Fisher’s exact test was used when cells have count = 0 or over 20% cells had expected count < 5.

**Table S5. IP-MS analysis of endogenous RNF138-associated protein complexes.**

| Description | Gene | Accession | M.W. | Spectral Count |
| --- | --- | --- | --- | --- |
| Peroxisome proliferator activated receptor interacting complex protein | PRIC295 | E1NZA1 | 293 kDa | 129 |
| Matrin-3 | MATR3 | P43243 | 95 kDa | 61 |
| Heterogeneous nuclear ribonucleoprotein U | HNRPU | Q00839 | 91 kDa | 46 |
| DNA mismatch repair protein Msh6 | MSH6 | P52701 | 153 kDa | 45 |
| ATP-dependent RNA helicase DDX24 | DDX24 | Q9GZR7 | 96 kDa | 44 |
| U2 snRNP-associated SURP motif-containing protein | U2SURP | O15042 | 118 kDa | 44 |
| RRP12-like protein OS=Homo sapiens | RRP12 | Q5JTH9 | 144 kDa | 44 |
| E3 ubiquitin-protein ligase TRIP12 | TRIPC | Q14669 | 220 kDa | 44 |
| E3 SUMO-protein ligase RanBP2 | RANBP2 | P49792 | 358 kDa | 44 |
| Poly [ADP-ribose] polymerase 1 | PARP1 | P09874 | 113 kDa | 43 |
| Nuclear pore complex protein Nup107 | NU107 | P57740 | 106 kDa | 36 |
| SWI/SNF complex subunit SMARCC2 | SMRCC2 | Q8TAQ2 | 133 kDa | 34 |
| Spermatogenesis-associated protein 5 | SPATA5 | Q8NB90 | 98 kDa | 29 |
| RANBP2-like and GRIP domain-containing protein 8 | RGPD8 | O14715 | 199 kDa | 22 |
| Splicing factor, proline- and glutamine-rich | SFPQ | P23246 | 76 kDa | 21 |
| MHC class II regulatory factor RFX1 | RFX1 | P22670 | 105 kDa | 18 |
| Bcl-2-associated transcription factor 1 | BCLAF1 | Q9NYF8 | 106 kDa | 17 |
| MSH2 protein | MSH2 | V9H019 | 91 kDa | 15 |
| Importin-4 | IPO4 | Q8TEX9 | 119 kDa | 13 |
| CDC2L2 protein (Fragment) | CDC2L2 | Q96CA8 | 52 kDa | 12 |
| Heat shock protein HSP 90-alpha | HS90AA1 | P07900 | 85 kDa | 11 |
| SNRNP200 protein (Fragment) | SNRNP200 | A4FU77 | 216 kDa | 9 |
| Pre-mRNA-processing-splicing factor 8 | PRPF8 | Q6P2Q9 | 273 kDa | 9 |
| Mov10, Moloney leukemia virus 10, homolog (Mouse), isoform CRA_a | MOV10 | Q5JR04 | 107 kDa | 9 |
| UDP-N-acetylglucosamine--peptide N-acetylglucosaminyltransferase 110 kDa subunit | OGT | O15294 | 117 kDa | 9 |
| MMS19 nucleotide excision repair protein homolog | MMS19 | Q96T76 | 113 kDa | 8 |
| DNA-dependent protein kinase catalytic subunit | PRKDC | P78527 | 469 kDa | 8 |
| Eukaryotic translation initiation factor 4 gamma 2 | EIF4G2 | H0Y3P2 | 98 kDa | 7 |
| PTCD1 protein (Fragment) | PTCD1 | Q3SYP6 | 53 kDa | 7 |
| Keratin, type I cytoskeletal 16 | KRT16 | P08779 | 51 kDa | 7 |
| Catenin alpha-2 | CTNNA2 | P26232 | 105 kDa | 7 |
| AP-1 complex subunit gamma-1 | AP1G1 | O43747 | 91 kDa | 6 |
| E3 ubiquitin-protein ligase HECTD1 | HECD1 | Q9ULT8 | 289 kDa | 6 |
| Nuclear factor-related to kappa-B-binding protein (Fragment) | NFRKB | E9PQ59 | 108 kDa | 6 |
| Ran-binding protein 6 | RANBP6 | O60518 | 125 kDa | 6 |
| COUP transcription factor 2 | NR2F2 | P24468 | 46 kDa | 5 |
| Serine/arginine repetitive matrix protein 2 | SRRM2 | Q9UQ35 | 299 kDa | 5 |
| Nesprin-2 | SYNE2 | G3V5X4 | 787 kDa | 5 |
| PRDM10 protein | PRDM10 | B7ZL72 | 121 kDa | 4 |
| PWWP domain-containing protein 2A | PWWP2A | Q96N64 | 82 kDa | 4 |
| Mdm2-binding protein | MTBP | Q96DY7 | 102 kDa | 4 |
| Transcriptional repressor CTCF | CTCF | P49711 | 83 kDa | 3 |
| Heterogeneous nuclear ribonucleoprotein M | HNRNPM | A0A087X0X3 | 78 kDa | 3 |
| XPO4 protein (Fragment) | XPO4 | Q86VC1 | 94 kDa | 3 |
| General transcription factor IIi isoform D (Fragment) | GTF2I | X5D2J9 | 108 kDa | 3 |
| E3 ubiquitin-protein ligase DZIP3 | DZIP3 | Q86Y13 | 139 kDa | 3 |
| NOP56 protein (Fragment) | NOP56 | A0PJ92 | 50 kDa | 3 |
| E3 SUMO-protein ligase ZNF451 | ZNF451 | E9PH99 | 119 kDa | 3 |
| Ras-responsive element-binding protein 1 (Fragment) | RREB1 | C9JU34 | 85 kDa | 3 |
| Double-strand-break repair protein rad21 homolog | RAD21 | O60216 | 72 kDa | 3 |
| PPFIBP1 protein (Fragment) | PPFIBP1 | Q05CN4 | 34 kDa | 3 |
| Nuclear pore complex protein Nup214 | NUP214 | P35658 | 213 kDa | 2 |
| Ubiquitin-like modifier-activating enzyme 5 | UBA5 | E7EQ61 | 42 kDa | 2 |
| Cell division cycle 2-like 5 (Cholinesterase-related cell division controller), isoform CRA_b | CDC2L5 | A0A024RA66 | 158 kDa | 2 |
| NIK and IKKβ binding protein | NIBP | MS180520 | 128 kDa | 1 |
| Potassium voltage-gated channel subfamily KQT member 2 (Fragment) | KCNQ2 | A0A0D9SFE0 | 17 kDa | 1 |
| RING finger protein 10 (Fragment) | RNF10 | H0YF69 | 15 kDa | 1 |
| BOP1 protein (Fragment) | BOP1 | Q6DKJ9 | 59 kDa | 1 |
| Histone H1.2 | HIST1H1C | P16403 | 21 kDa | 1 |
| DNA replication licensing factor MCM4 (Fragment) | MCM4 | E5RG31 | 32 kDa | 1 |
| E3 ubiquitin-protein ligase HACE1 (Fragment) | HACE1 | H0YAU8 | 34 kDa | 1 |
| PWWP domain-containing protein MUM1 | MUM1 | Q2TAK8 | 79 kDa | 1 |
| Nuclear export mediator factor NEMF | NEMF | G3V547 | 14 kDa | 1 |
| eIF2AK2 protein (Fragment) | EIF2AK2 | Q05CP4 | 41 kDa | 1 |
| Ataxin-10 | ATXN10 | A0A1W2PQD2 | 45 kDa | 1 |
| MAP kinase-activating death domain protein | MADD | F8W8U2 | 49 kDa | 1 |
| NEK9 protein (Fragment) | NEK9 | Q6PKF2 | 34 kDa | 1 |
| CASP8-associated protein 2 | CASP8AP2 | A0A087WTW5 | 221 kDa | 1 |
| Nucleolar protein 11 (Fragment) | NOL11 | J3QLQ6 | 21 kDa | 1 |
| Nipped-B protein | NIPBL | Q6IEH8 | 316 kDa | 1 |
| TJP3 protein (Fragment) | TJP3 | Q2VPE5 | 101 kDa | 1 |
| IASPP short isoform | PPP1R13L | A7YME7 | 44 kDa | 1 |
| RRP1B protein (Fragment) | RRP1B | Q6PJM8 | 47 kDa | 1 |
| Ataxin 3 variant m | ATXN3 | D3VVE1 | 35 kDa | 1 |
| Small G protein-signaling modulator 1 | SGSM1 | A0A087X241 | 123 kDa | 1 |
| MUC12 protein (Fragment) | MUC12 | A1L198 | 65 kDa | 1 |
| MCM10 minichromosome maintenance deficient 10 (S. cerevisiae), isoform CRA_b | MCM10 | Q5T670 | 96 kDa | 1 |
| Mitogen-activated protein kinase kinase kinase 6 | MAP3K6 | O95382 | 143 kDa | 1 |
| Scm-like with four MBT domains protein 1 | SFMBT1 | Q9UHJ3 | 98 kDa | 1 |
| TRPM8 channel-associated factor 1 | TCAF1 | Q9Y4C2 | 102 kDa | 1 |
| Keratin, type I cytoskeletal 39 | KRT39 | Q6A163 | 56 kDa | 1 |
| Nucleolar pre-ribosomal-associated protein 1 | URB1 | O60287 | 254 kDa | 1 |
| PDZ domain-containing protein 8 | PDZD8 | Q8NEN9 | 128 kDa | 1 |
| NAD-dependent protein deacetylase sirtuin-2 | SIRT2 | F8WBT6 | 11 kDa | 1 |
| Activating signal cointegrator 1 complex subunit 3 | ASCC3 | Q8N3C0 | 251 kDa | 1 |
| DDB1- and CUL4-associated factor 1 | DCAF1 | Q9Y4B6 | 169 kDa | 1 |
| Mitogen-activated protein kinase kinase kinase 1 | MAP3K1 | Q13233 | 164 kDa | 1 |
| Neurobeachin-like protein 1 | NBEAL1 | Q6ZS30 | 307 kDa | 1 |
| Retinoblastoma-like protein 1 | RBL1 | P28749 | 121 kDa | 1 |
| Pericentrin | PCNT | O95613 | 378 kDa | 1 |
| Mitotic checkpoint serine/threonine-protein kinase BUB1 beta | BUB1B | O60566 | 119 kDa | 1 |
| M-phase phosphoprotein 8 | MPHOSPH8 | Q99549 | 97 kDa | 1 |
| Putative GED domain-containing protein DNM1P46 | DNM1P46 | Q6ZS02 | 24 kDa | 1 |
| Alternative protein TMEM105 | TMEM105 | L8EAT6 | 8 kDa | 1 |
| E3 ubiquitin-protein ligase RFWD3 | RFWD3 | Q6PCD5 | 85 kDa | 1 |
| Phospholipase DDHD1 | DDHD1 | Q8NEL9 | 100 kDa | 1 |
| Mediator of RNA polymerase II transcription subunit 13 | MED13 | Q9UHV7 | 239 kDa | 1 |

**Table S6. Summary of patient-derived CRC xenograft and corresponding clinical data.**

| ID | Line | Gender | Diagnosis | Overall tumor stage | RNF138 | pp65 | Passage  number |
| --- | --- | --- | --- | --- | --- | --- | --- |
| Patient-1 | PDX^high1^ | Male | CRC | T4N2M | high | low | 4 |
| Patient-2 | PDX^high2^ | Male | CRC | T4N1M | high | low | 4 |
| Patient-3 | PDX^low1^ | Female | CRC | T3N0M1 | low | high | 4 |
| Patient-4 | PDX^low2^ | Male | CRC | T4N2M1 | low | high | 4 |

**Table S7. key resources table.**

| REAGENT or RESOURCE | SOURCE | IDENTIFIER | | | |
| --- | --- | --- | --- | --- | --- |
| Antibodies | | |  |  |  |
| Rat monoclonal anti-RNF138 | Made in our lab | N/A | | | |
| Rabbit polyclonal anti-NIBP | Proteintech | CAT# 16014-1-AP | | | |
| Rabbit polyclonal anti-TRAPPC3 | Proteintech | CAT# 15555-1-AP | | | |
| Rabbit monoclonal anti-phospho-NF-κB p65 (Ser536) | Cell Signaling Technology  Abcam | CAT# 3033  CAT# ab86299 | | | |
| Rabbit monoclonal anti-NF-κB p65 | Cell Signaling Technology | CAT# 8242 | | | |
| Rabbit monoclonal anti-phospho-IKKα/β (Ser176/180) | Cell Signaling Technology | CAT# 2697 | | | |
| Rabbit monoclonal anti-IKKβ | Cell Signaling Technology | CAT# 8943 | | | |
| Mouse monoclonal anti-GAPDH | Proteintech | CAT# 60004-1-Ig | | | |
| Rabbit recombinant anti-ICAM1 | Abcam | CAT# ab53013 | | | |
| Rabbit monoclonal anti- PTGS2 | Cell Signaling Technology | CAT# 12282 | | | |
| Mouse monoclonal anti-Flag | Sigma-Aldrich | CAT# F1804 | | | |
| Rabbit monoclonal anti-HA | Cell Signaling Technology | CAT# 3724 | | | |
| Rabbit monoclonal anti-Histone H3 | Cell Signaling Technology | CAT# 4499 | | | |
| Rabbit monoclonal anti-Ki67 | Abcam | CAT# ab16667 | | | |
| Rabbit monoclonal anti-phospho-Histone H2AX (Ser139) | Cell Signaling Technology | CAT# 9718 | | | |
| Mouse monoclonal anti-STAT3 | Cell Signaling Technology | CAT# 9139 | | | |
| Rabbit monoclonal anti-β-Catenin | Cell Signaling Technology | CAT# 8480 | | | |
| Normal rabbit IgG | Millipore | CAT# 12-370 | | | |
| Normal rat IgG | Santa Cruz Biotechnology | CAT# sc-2026 | | | |
| Goat Alexa Fluor Plus 647 anti-Rabbit IgG (H+L) | Invitrogen | CAT# A32733 | | | |
| Donkey DyLight 594 anti-Mouse IgG (H+L) | Invitrogen | CAT# SA5-10168 | | | |
| Goat Alexa Fluor 488 anti-Rat IgG (H+L) | Invitrogen | CAT# A-11006 | | | |
| Goat anti-Rabbit IgG-HRP (H+L) | Zhongshan Golden Bridge Biotechnology | CAT# ZB-2301 | | | |
| Goat anti-Mouse IgG-HRP (H+L) | Zhongshan Golden Bridge Biotechnology | CAT# ZB-2305 | | | |
| Goat anti-Rat IgG-HRP (H+L) | Zhongshan Golden Bridge Biotechnology | CAT# ZB-2307 | | | |
| Biological Samples | | | |  |  |
| TCGA patient clinic data | TCGA data portal | https://tcga-data.nci.nih.gov/tcga/ | | | |
| Tumor and adjacent normal tissues for qRT-PCR, immunoblotting and immunostaining | Cancer Hospital, Chinese Academy of Medical Sciences | N/A | | | |
| 420 TMAs | Cancer Hospital, Chinese Academy of Medical Sciences | N/A | | | |
| PDX | Cancer Hospital, Chinese Academy of Medical Sciences | N/A | | | |
| Chemical, Peptides and Recombinant Proteins | | | |  |  |
| Dextran Sulfate Sodium | MP Biomedicals | CAT# 215676080 | | | |
| Azoxymethane | Sigma-Aldrich | CAT# A5486 | | | |
| 4% [paraformaldehyde](javascript:;) | Servicebio | CAT# G1101 | | | |
| Dulbecco’s Modified Eagle Medium | Gibco | CAT# 11965092 | | | |
| Iscove Modified Dulbecco Medium | Gibco | CAT# 12440046 | | | |
| Fetal Bovine Serum | Gibco | CAT# 10099141C | | | |
| Penicillin-Strptomycin | Gibco | CAT# 15070063 | | | |
| Lipofectamine 2000 | Invitrogen | CAT# 11668030 | | | |
| Lipofectamine 3000 | Invitrogen | CAT# L3000075 | | | |
| Lipofectamine RNAiMAX | Invitrogen | CAT# 13778150 | | | |
| Dispase | Gibco | CAT# 17105-041 | | | |
| HEPES | ThermoFisher Scientific | CAT# [15630106](https://www.thermofisher.com/order/catalog/product/15630106) | | | |
| GlutaMAX supplement | Gibco | CAT# 35050-079 | | | |
| N2 supplement | Invitrogen | CAT# 17502-048 | | | |
| B27 supplement | Invitrogen | CAT# 17504-044 | | | |
| Collagenase type IV | Gibco | CAT# 17104-019 | | | |
| N-acetylcysteine | Sigma-Aldrich | CAT# A9165-5G | | | |
| Epidermal Growth Factor (EGF), Murine, Natural | Invitrogen | CAT# 53003-018 | | | |
| Advanced Dulbecco's Modified Eagle Medium/F12 | Invitrogen | CAT# 12634010 | | | |
| Matrigel Basement Membrane Matrix | BD Biosciences | CAT# 356237 | | | |
| Matrigel Matrix Basement Membrane | Corning | CAT# 354234 | | | |
| RIPA buffer | Cell Signaling Technology | CAT# 9806S | | | |
| cOmplete™, Mini, EDTA-free Protease Inhibitor Cocktail | Roche | CAT# 11836170001 | | | |
| PhosStop, 20 tablets, EASYpack | Roche | CAT# 4906837001 | | | |
| Tween 20 | Sigma-Aldrich | CAT# P9416 | | | |
| polyvinylidene difluoride membrane | GE Healthcare Life Sciences | CAT# RPN303F | | | |
| TRIzol Reagent | Invitrogen | CAT# 15596018 | | | |
| PowerUp^TM^ SYBR^TM^ Green Master Mix | Applied Biosystems | CAT# A25742 | | | |
| Coverslip | NEST | CAT# 801008 | | | |
| Triton-X 100 | Sigma-Aldrich | CAT# T8787 | | | |
| Mounting Medium with DAPI | Zhongshan Golden Bridge Biotechnology | CAT# ZLI-9557 | | | |
| Normal goat serum | Zhongshan Golden Bridge Biotechnology | CAT# ZLI-9021 | | | |
| Cry-Gel Embedding Medium | Leica | CAT# 39475237 | | | |
| EDTA antigen retrival solution (pH=9.0) | Zhongshan Golden Bridge Biotechnology | CAT# ZLI-9069 | | | |
| Citrate antigen retrival solution (pH = 6.0) | Zhongshan Golden Bridge Biotechnology | CAT# ZLI-9064 | | | |
| N-His-pET-28a vector | Gift from Pro. Zhang Xiaodong Lab. | NA | | | |
| p3 × Flag CMV14 vector | Gift from Pro. Zhang Xiaodong Lab. | NA | | | |
| N-HA-pcDNA 6.0 vector | Gift from Pro. Zhang Xiaodong Lab. | NA | | | |
| LentiCRISP-v2-bsd vector | Addgene | CAT# 52961 | | | |
| Plasmids psPAX2 | Addgene | CAT# 12260 | | | |
| Plasmids pMD2.G | Addgene | CAT# 12259 | | | |
| PCDH-EF1-MCS-T2A-puro vector | Addgene | CAT# 72263 | | | |
| IPTG | Sigma-Aldrich | CAT# I6758 | | | |
| Transetta (DE3) [competent](C:/Users/lulu/AppData/Local/youdao/dict/Application/8.9.6.0/resultui/html/index.html#/javascript:;) [cell](C:/Users/lulu/AppData/Local/youdao/dict/Application/8.9.6.0/resultui/html/index.html#/javascript:;) | TransGen Biotech | CAT# CD801-02 | | | |
| Amicon Ultra | Millipore | CAT# UFC901096 | | | |
| HAT Media Supplement (50×) | Sigma-Aldrich | CAT# H0262 | | | |
| Kpn I | Takara | CAT# 1068A | | | |
| BamH I | Takara | CAT# 1010A | | | |
| EcoR 1 | Takara | CAT# 1040B | | | |
| Blasticidin S HCl | Selleckchem | CAT# S7419 | | | |
| Puromycin | MP Biomedicals | CAT# 194539 | | | |
| Progein G PLUS agarose beads | Santa Cruz Biotechnology | CAT# sc-2002 | | | |
| Protein A Agarose | Santa Cruz Biotechnology | CAT# sc-2001 | | | |
| Dynabeads^TM^ Protein G | Invitrogen | CAT# 10004D | | | |
| Dimethyl sulfoxide | Sigma-Aldrich | CAT# D2650 | | | |
| Corn oil | Solarbio | CAT# C7030 | | | |
| SC75741 | Selleckchem | CAT# S7273 | | | |
| IKK16 (IKK Inhibitor Ⅶ) | Selleckchem | CAT# S2882 | | | |
| BAY11-7082 | Selleckchem | CAT# S2913 | | | |
| Bortezomib (PS-341) | Selleckchem | CAT# S1013 | | | |
| Critical Commercial Assays | | | | |  |
| RNeasy Mini Kit | Qiagen | CAT# 74104 | | | |
| RevertAid First Strand cDNA Synthesis Kit | ThermoFisher Scientific | CAT# K1622 | | | |
| BCA protein assay kit | Pierce Biotechnology | CAT# 23225 | | | |
| ECL detection system | Millipore | CAT# WBULS0500 | | | |
| Polink-2 plus® Polymer HRP Detection System For Rabbit Primary Antibody | Zhongshan Golden Bridge Biotechnology | CAT# PV-9001 | | | |
| Polink-2 plus® Polymer HRP Detection System For Mouse Primary Antibody | Zhongshan Golden Bridge Biotechnology | CAT# PV-9002 | | | |
| Polink-2 plus® Polymer HRP Detection System For Rat Primary Antibody | Zhongshan Golden Bridge Biotechnology | CAT# PV-9004 | | | |
| DAB detection kit | Zhongshan Golden Bridge Biotechnology | CAT# [ZLI-901](https://www.so.com/link?m=a13eIrlqTBpQPR4pV4ID6UIhmTW1Qep71AUpgDgPs4Bmu32V63JZiTSEzvvJDBNyMeHJ9jOYzO16%2BnFYzvhMkwiXsEU4A%2Fmh8rp9W%2FOk7JScj4%2FEoJ51js6Ez8JUPRiTExv8DytVk0zhickllXZjm502PsO27WtxmUrnXBP9tiP8OdQHEWzcSK46trgMwpl%2FltvIEJOA9gBVsyyN38Rb%2F1NsPp1zDGb9ArVX0GuRK0NrhJQhCoD4lmtnWcc7FTbpiulpaMG51dEzQwHkpYNX7DIV%2Fhlo%3D)8 | | | |
| Mycoplasma Detection Kit-QuickTest | Biotool | CAT# B39035 | | | |
| Cell Counting Kit-8 | Dojindo | CAT# CK04 | | | |
| Quick-Change Site-Directed Mutagenesis kit | Stratagene | CAT# 200518 | | | |
| Colloidal Blue Staining Kit | Invitrogen | CAT# LC6025 | | | |
| Duolink In Situ kit | Sigma-Aldrich | CAT# DUO92008 | | | |
| Duolink PLA oligonucleotides （MINUS） | Sigma-Aldrich | CAT# DUO92010 | | | |
| Experimental Models: Organisms/Strains | | | | |  |
| Mouse: RNF138^fl/fl^ mice | Generated by our lab | N/A | | | |
| Mouse: RNF138^-/-^ mice | Generated by our lab | N/A | | | |
| Mouse: BALB/c nude mice | Beijing Vital River Laboratory Animal Technology | N/A | | | |
| Mouse: NOD/SCID mice | Beijing Vital River Laboratory Animal Technology | N/A | | | |
| Cell line: HCT116 | Cell Resource Center of Peking Union Medical College | N/A | | | |
| Cell line: RKO | Cell Resource Center of Peking Union Medical College | N/A | | | |
| Cell line: HEK293T | Cell Resource Center of Peking Union Medical College | N/A | | | |
| Oligonucleotides | | | | |  |
| Control-siRNA oligo | GenePharma company | N/A | | | |
| siRNF138 #1: CCUGUGUCAAGAAUCAAAU | GenePharma company | N/A | | | |
| siRNF138 #2: GGAUCACUGUAACAGUAAU | GenePharma company | N/A | | | |
| siRNF138 #3: CUUCAGCUAGAUGAAGAAA | GenePharma company | N/A | | | |
| siNIBP #1: GCUGCUGCGUUCUGUGAAUTT | GenePharma company | N/A | | | |
| siNIBP #2: GCCUUAGCCCUGAAGACAUTT | GenePharma company | N/A | | | |
| siNIBP #3: GCAUGGAAGCAUCAGAAUUTT | GenePharma company | N/A | | | |
| sgRNF138: CAAAACGCCCGTGCGGACCA | Invitrogen | N/A | | | |
| RNF138--p3 X Flag-CMV14-FL-F: TTCATCGATAGATCTGATATCGGTACCATGGCCGAGGACCTCTCTGC | Invitrogen | N/A | | | |
| RNF138--p3 X Flag-CMV14--FL-R: ATGGTCTTTGTAGTCAGCCCGGGATCCGATGTTTACTTGAAAAGATTCTTC | Invitrogen | N/A | | | |
| RNF138--p3 X Flag-CMV14--ΔRING-F: TACACCGAAGATGATTTCTACGGAAATGTGACTAGAAGAGAG | Invitrogen | N/A | | | |
| RNF138--p3 X Flag-CMV14--ΔRING-R: CTCTCTTCTAGTCACATTTCCGTAGAAATCATCTTCGGTGTA | Invitrogen | N/A | | | |
| RNF138--p3 X Flag-CMV14--ΔZNF1-F: ATGAGGAAGTTTTCTGGTAGCAAGAAGTATCAGGATGAATAT | Invitrogen | N/A | | | |
| RNF138--p3 X Flag-CMV14--ΔZNF1-R: ATATTCATCCTGATACTTCTTGCTACCAGAAAACTTCCTCAT | Invitrogen | N/A | | | |
| RNF138--p3 X Flag-CMV14--ΔZNF2-F: TCTGGTCATCCTACTTTTAAGCTATTTCAGATAGTTCCTGTG | Invitrogen | N/A | | | |
| RNF138--p3 X Flag-CMV14--ΔZNF2-R: CACAGGAACTATCTGAAATAGCTTAAAAGTAGGATGACCAGA | Invitrogen | N/A | | | |
| RNF138--p3 X Flag-CMV14--ΔZNF3-F: TTTCAGATAGTTCCTGTGACACAATTTGATTATGGAGAATTT | Invitrogen | N/A | | | |
| RNF138--p3 X Flag-CMV14--ΔZNF3-R: AAATTCTCCATAATCAAATTGTGTCACAGGAACTATCTGAAA | Invitrogen | N/A | | | |
| RNF138--p3 X Flag-CMV14--ΔUIM-F: TTTGTGAATCTTCAGCTAGATAACATCGGATCCCGGGCTGAC | Invitrogen | N/A | | | |
| RNF138--p3 X Flag-CMV14--ΔUIM-R: GTCAGCCCGGGATCCGATGTTATCTAGCTGAAGATTCACAAA | Invitrogen | N/A | | | |
| RNF138--p3 X Flag-CMV14--C18A-F: TACACCGAAGATGATTTCTACGCCCCCGTCTGTCAGGAGGTGCTC | Invitrogen | N/A | | | |
| RNF138--p3 X Flag-CMV14--C18A-R: GAGCACCTCCTGACAGACGGGGGCGTAGAAATCATCTTCGGTGTA | Invitrogen | N/A | | | |
| RNF138--p3 X Flag-CMV14--C54A-F: ATGAGGGAAAGCGGAGCACATGCTCCCCTATGTCGTGGAAATGTG | Invitrogen | N/A | | | |
| RNF138--p3 X Flag-CMV14--C54A-R: CACATTTCCACGACATAGGGGAGCATGTGCTCCGCTTTCCCTCAT | Invitrogen | N/A | | | |
| NIBP--p3 X Flag-CMV14-FL-F: AAG CTT GCG GCC GCG AAT TC ATGAGCGTCCCTGACTACAT | Invitrogen | N/A | | | |
| NIBP--p3 X Flag-CMV14-FL-R: ATGGTCTTTGTAGTCAGCCCGGGATCCGGCCTGCGCCTCCAGGGCAC | Invitrogen | N/A | | | |
| NIBP--p3 X Flag-CMV14-FL-F: AAG CTT GCG GCC GCG AAT TC ATGAGCGTCCCTGACTACAT | Invitrogen | N/A | | | |
| NIBP--p3 X Flag-CMV14-FL-R: ATGGTCTTTGTAGTCAGCCC GGGATCCGGC CTGCGCCTCCAGGGCAC | Invitrogen | N/A | | | |
| NIBP--N-HA-pcDNA 6.0 vector-FL-F: AAG CTT GCG GCC GCG AAT TC ATGAGCGTCCCTGACTACAT | Invitrogen | N/A | | | |
| NIBP--N-HA-pcDNA 6.0 vector-FL-R: ATGGTCTTTG TAGTCAGCCCGGGATCCGGCCTGCGCCTCCAGGGCAC | Invitrogen | N/A | | | |
| NIBP--p3 X Flag-CMV14-ΔN359-F: AAGCTTGCGGCCGCG AAT TCATG CGG AGCATGGAA GCATCAGAA | Invitrogen | N/A | | | |
| NIBP--p3 X Flag-CMV14-ΔN359-R: ATGGTCTTTGTAGTCAGCCC GGGATCCGGCCTGCGCCTCC AGGGCAC | Invitrogen | N/A | | | |
| NIBP--p3 X Flag-CMV14-ΔC480-F: AAGCTTGCGGCCGCGAATTCATGAGCGTCCCTGACTACAT | Invitrogen | N/A | | | |
| NIBP--p3 X Flag-CMV14-ΔC480-R: ATGGTCTTTGTAGTCAGCCC GGGATCCCAC ACCGAAGACCGTGGTATG | Invitrogen | N/A | | | |
| NIBP--p3 X Flag-CMV14-1-266-F: AAGCTTGCGGCCGCGAATTCATGAGCGTCCCTGACTACAT | Invitrogen | N/A | | | |
| NIBP--p3 X Flag-CMV14-1-266-R: ACAGACGGCTCGACTTCTACACTCTTCCCACCAGTTCCAC | Invitrogen | N/A | | | |
| NIBP--p3 X Flag-CMV14-882-1149-F: GTGGAACTGGTGGGAAGAGTGTAGAA GTCGAGCCGTCTGT | Invitrogen | N/A | | | |
| NIBP--p3 X Flag-CMV14-882-1149-R: ATGGTCTTTGTAGTCAGCCCGGGATCCGGCCTGCGCCTCCAGGGCAC | Invitrogen | N/A | | | |
| qPCR_RNF138_F: CAGACAGCGTTTACTGGATCAC | Invitrogen | N/A | | | |
| qPCR_RNF138_R: TGGTAATCTGGCTAGGATCTCC | Invitrogen | N/A | | | |
| qPCR_NIBP_F: TCTACCCCAACTACGAGGACT | Invitrogen | N/A | | | |
| qPCR_NIBP_R: TGGCACCGCTTCTTGTAATGT | Invitrogen | N/A | | | |
| qPCR_CXCL1_F: AGTCATAGCCACACTCAAGAATGG | Invitrogen | N/A | | | |
| qPCR_CXCL1_R: GATGCAGGATTGAGGCAAGC | Invitrogen | N/A | | | |
| qPCR_NFκB1_F: AACAGAGAGGATTTCGTTTCCG | Invitrogen | N/A | | | |
| qPCR_NFκB1_R: TTTGACCTGAGGGTAAGACTTCT | Invitrogen | N/A | | | |
| qPCR_IκBα_F: ACCTGGTGTCACTCCTGTTGA | Invitrogen | N/A | | | |
| qPCR_IκBα_R: CTGCTGCTGTATCCGGGTG | Invitrogen | N/A | | | |
| qPCR_IL8_F: ACTGAGAGTGATTGAGAGTGGAC | Invitrogen | N/A | | | |
| qPCR_IL8_R: AACCCTCTGCACCCAGTTTTC | Invitrogen | N/A | | | |
| qPCR_PTGS2_F: CTGGCGCTCAGCCATACAG | Invitrogen | N/A | | | |
| qPCR_PTGS2_R: CGCACTTATACTGGTCAAATCCC | Invitrogen | N/A | | | |
| qPCR_ICAM1_F: ATGCCCAGACATCTGTGTCC | Invitrogen | N/A | | | |
| qPCR_ICAM1_R: GGGGTCTCTATGCCCAACAA | Invitrogen | N/A | | | |
| qPCR_ ACTB_F: CATGTACGTTGCTATCCAGGC | Invitrogen | N/A | | | |
| qPCR_ ACTB_R: CTCCTTAATGTCACGCACGAT | Invitrogen | N/A | | | |
| Deposited Data | | | | |  |
| RNA sequencing data | This study | GSE144051 | | | |
| RNF138 antibody | This study | 202010452272.0 | | | |
| Recombinant DNA | | | | |  |
| Flag-RNF138-FL | This study | N/A | | | |
| Flag-RNF138-ΔRING | This study | N/A | | | |
| Flag-RNF138-ΔZNF1-3 | This study | N/A | | | |
| Flag-RNF138-ΔUIM | This study | N/A | | | |
| Flag-RNF138-C18A/C54A | This study | N/A | | | |
| HA-NIBP-FL | This study | N/A | | | |
| Flag-NIBP-FL | This study | N/A | | | |
| Flag-NIBP-ΔN359 | This study | N/A | | | |
| Flag-NIBPΔC480 | This study | N/A | | | |
| Flag-NIBPΔM616 | This study | N/A | | | |
| Software and Algorithms | | | | |  |
| GraphPad Prism 8.0 Software | GraphPad Software | https://www.graphpad.com/ | | | |
| SPSS Statistics 20.0 | IBM | N/A | | | |
| Origin 2019b | OriginLab | N/A | | | |
| Image J | NIH | https://imagej.nih.gov/ij/ | | | |
| Adobe Illustrator CS6 | Adobe | N/A | | | |
| Adobe Photoshop CS6 | Adobe | N/A | | | |
| Other | | | | |  |
| NanoDrop 2000 | Thermo Fisher Scientific | N/A | | | |
| Olympus BX43 light microscope/ Olympus  DP27 camera | Olympus | N/A | | | |
| ZEN blue | Carl Zeiss | N/A | | | |
| ZEN black | Carl Zeiss | N/A | | | |
| CFX Connect Real-Time Detection System | Bio-Rad | N/A | | | |
